# Optimal national prioritization policies for hospital care during the SARS-CoV-2 pandemic

**DOI:** 10.1038/s43588-021-00111-1

**Published:** 2021-08-13

**Authors:** Josh C. D’Aeth, Shubhechyya Ghosal, Fiona Grimm, David Haw, Esma Koca, Krystal Lau, Stefano Moret, Dheeya Rizmie, Sarah R. Deeny, Pablo N. Perez-Guzman, Neil Ferguson, Katharina Hauck, Peter C. Smith, Giovanni Forchini, Wolfram Wiesemann, Marisa Miraldo

**Affiliations:** 1 MRC Centre for Global Infectious Disease Analysis and WHO Collaborating Centre for Infectious Disease Modelling, School of Public Health, Imperial College London, London, UK; 2 Abdul Latif Jameel Institute for Disease and Emergency Analytics (J-IDEA), School of Public Health, Imperial College London, London, UK; 3 Department of Analytics, Marketing and Operations, Imperial College Business School, Imperial College London, London, UK; 4 The Health Foundation, London, UK; 5 Department of Economics and Public Policy, Imperial College Business School, Imperial College London, London, UK; 6 Centre for Health Economics and Policy Innovation, Imperial College Business School, Imperial College London, London, UK; 7 Centre for Health Economics, University of York, York, UK; 8 Umeå School of Business, Economics and Statistics, Umeå University, Umeå, Sweden

**Keywords:** Health care economics, Health policy, Health services, SARS-CoV-2

## Abstract

In response to unprecedented surges in the demand for hospital care during the SARS-CoV-2 pandemic, health systems have prioritized patients with COVID-19 to life-saving hospital care to the detriment of other patients. In contrast to these ad hoc policies, we develop a linear programming framework to optimally schedule elective procedures and allocate hospital beds among all planned and emergency patients to minimize years of life lost. Leveraging a large dataset of administrative patient medical records, we apply our framework to the National Health Service in England and show that an extra 50,750–5,891,608 years of life can be gained compared with prioritization policies that reflect those implemented during the pandemic. Notable health gains are observed for neoplasms, diseases of the digestive system, and injuries and poisoning. Our open-source framework provides a computationally efficient approximation of a large-scale discrete optimization problem that can be applied globally to support national-level care prioritization policies.

## Abstract (web summary)

Countries are using hospital admission policies that prioritize patients with COVID-19 during the pandemic. The authors propose an alternative open-source framework to optimally schedule hospital care for all diseases and patients that can save life years overall.

## Main

Health systems worldwide are struggling to provide hospital treatment during the surges in demand for emergency care caused by the severe acute respiratory syndrome coronavirus 2 (SARS-CoV-2) pandemic, despite efforts to increase capacity^1–4^. To manage demand, many countries have prioritized patients with coronavirus disease 2019 (COVID-19), canceled elective (that is, planned) procedures^5–7^ and rationed access to life-saving care for all patients^3,4^. For example, policies in Italy, in March 2020, involved prioritizing intensive care to patients with COVID-19 above 70 years who previously had no more than one admission per year for a chronic illness (for example, exacerbated chronic obstructive pulmonary disease, advanced neoplasms and congestive heart failure)^8^. In England, the cancellation of non-urgent elective surgeries after 17 March 2020 was combined with the prioritization to critical care (CC) of those with high capacity to benefit as signaled by a low frailty score^5,6^. Even though several vaccines are now available, as the pandemic progresses, hospitals are still challenged by capacity constraints to schedule elective surgeries^3,4,9^.

Policies that prioritize patients with COVID-19 over other patients with greater capacity to benefit may result in immediate or delayed deaths and morbidity among both non-COVID-19 patients and patients with COVID-19 and increase the financial burden on health systems as delays in planned treatments may accelerate disease progression and the need for more costly interventions later^10,11^. Also, when implemented, these policies generate a backlog of non-COVID-19 patients in need of care^12,13^ that require prioritization rules that differ from pre-pandemic ones in order to be managed, since heterogeneity in disease progression over the postponement period might change their relative priority when compared with other patients. In England, the National Health Service (NHS) Confederation has projected waiting lists to reach 9.8 million by the end of 2021^14^, highlighting how essential it is to identify ways to prioritize care and prevent hospitals from being overwhelmed by the various constraints posed by the pandemic^15^.

The optimal scheduling of hospital care has been studied albeit focusing on specific diseases^16,17^, types of care^18–20^ or individual hospitals^21^. These studies deploy methodologies that allow for a faithful modeling of the operations of an individual hospital or a specific care setting and thus enable prioritization of subgroups of patients, but they do not scale to the health system of an entire country and all patients in need of care, which is the focus of our paper.

In many health systems worldwide, hospital scheduling relies on a set of prioritization rules, which seldom look at the entirety of the health system and therefore lead to suboptimal decisions. The need to mitigate this gap in evidence has been explicitly raised during the pandemic. In England, the Nuffield Council on Bioethics issued a national statement on the need for clearer and “nationally developed and coordinated guidance on how decisions about the allocation of constrained resources should be made,”^22^ highlighting also the need for prioritization to consider all disease groups and their capacity to benefit. Therefore, in contrast to the approach followed in the literature, we propose a framework to optimally schedule elective care and allocate hospital capacity to elective and emergency patients in general and acute (G&A) and CC across all disease areas while accounting for national capacity and needs.

Our approach relies on a linear programming (LP) formulation of the problem, which we present in abridged form in Methods and in its entirety in the Supplementary Information.

We apply our model to the NHS in England between 2 March 2020 and 1 March 2021, with the aim of minimizing years of life lost (YLL) under alternative scenarios. These consider capacity constraints, demand for emergency care and projected COVID-19 emergencies under a set of pandemic trajectories that reflect varying stringency of control strategies, projected with a susceptible–exposed–infected–recovered (SEIR) model of SARS-CoV-2 transmission. We compare the outcomes of our optimized schedules to those of a set of simulated prioritization policies that reflect those implemented by various health systems during the pandemic, including: (1) postponement of elective procedures; (2) prioritization to CC based on frailty; and (3) re-scheduling patients to elective care using pre-pandemic prioritization rules.

## Results

### Model overview

**Fig. 1:**
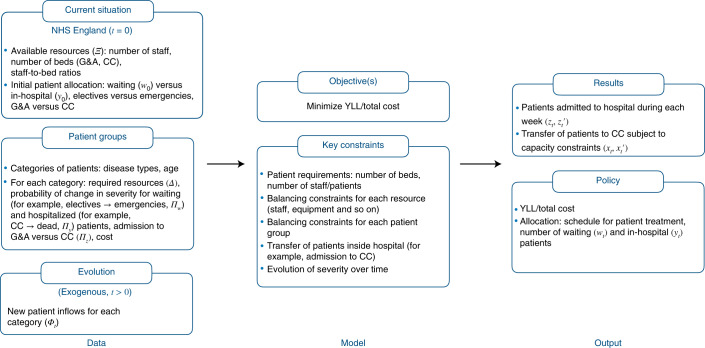
Input–output overview of the LP model formulation for the OS. Inputs of the model are (1) the initial situation (at *t* = 0) in terms of the available resources (*Ξ*) and the current allocation of patients (waiting versus in-hospital patients, in CC versus G&A, and so on). Patients are divided into different patient groups and subdivided on the basis of severity. For each subgroup, we provide as inputs (2) their resource requirements (*Δ*) as well as transition matrices (*Π*) representing the probabilities of endogenous transfers of patients between severity groups (for example, patients needing emergency care while waiting for elective care, or patients in G&A requiring CC). For *t* > 0, based on the scenarios we are investigating (for example, lockdown), we observe (3) new exogenous inflows of patients (*Φ*). During each week, the model optimizes the allocation of patients, that is, how many patients of each group to admit to hospital ($z_t,z_t^\prime$) as well as the in-hospital transfers of patients.

Optimized schedules (OS) are formulated with a deterministic LP model that optimally schedules the admission of patients to hospital across all diseases and nationally. Specifically, the key decision variables of the model are which patients to admit and when (admission scheduling), and which patients to allocate to CC in case of capacity shortages. In the OS, emergency patients are always admitted if capacity is available, while elective admissions are scheduled weekly over the 52-week horizon with the objective of minimizing YLL. Figure 1 offers a conceptual input–output overview of the LP formulation.

When demand exceeds capacity, OS ration care. First, patients in need of elective care remain on the waiting list. Second, patients requiring CC are treated in G&A (with different transition probabilities) until CC capacity becomes available. Third, during the peaks of the pandemic and under some scenarios, emergency capacity is not sufficient to accommodate all patients. Given that we cannot rely on pre-pandemic data to estimate the implications of denied emergency care, our analysis considers two boundary cases. Our LP model conservatively assumes that all patients who are denied emergency admission die (upper-bound case). Alternatively, we use the solution of our LP model but re-evaluate the YLL under the assumption that the denied patients’ probabilities and outcomes remain as if they would be cared for in hospital (lower-bound case). By modeling both extreme cases, we fully characterize the spectrum of reasonable outcomes.

We compare our OS with standard policies (SP) that simulate prioritization policies implemented in England during the pandemic. SP manage surges in demand by postponing elective admissions. Patients with postponed procedures are kept waiting and admitted once there is capacity according to a first-in-first-out rule, that is, following an order that preserves their assigned priority when care was originally scheduled. Different disease groups are admitted in equal proportions. Under SP, CC is prioritized for non-frail patients. All other assumptions are as in OS.

We model four SP informed by elective care postponements and actual admission policies in England. SP1 and SP3 assume that elective procedures were canceled between 17 March and 29 April 2020 (weeks 3–8), as actually occurred^7^. SP2 and SP4 additionally trigger cancellations anytime over the intervention horizon when the projected COVID-19 incidence exceeds specific trigger points. SP1 and SP2 assume 100%, SP3 and SP4 75% elective cancellations.

The incremental cost-effectiveness ratio (ICER, a standard metric used by policymakers to decide on interventions to implement in health systems) of OS versus SP is calculated across all disease groups and ages as (where YLG is years of life gained):

$${\mathrm{ICER}} = \frac{{{\Delta}{\mathrm{Costs}}}}{{{\Delta}{\mathrm{YLG}}}} = \frac{{{\mathrm{Cost}}_{{\mathrm{OS}}} - {\mathrm{Cost}}_{{\mathrm{SP}}}}}{{{\mathrm{YLL}}_{{\mathrm{SP}}} - {\mathrm{YLL}}_{{\mathrm{OS}}}}}$$

### Model data

To forecast non-COVID-19 elective and emergency needs (CC and G&A), we use administrative patient medical records (Hospital Episode Statistics^23^ (HES)) from all public acute hospitals in England between January 2015 and February 2020. To model care pathways of hospital patients with COVID-19, we use medical records from 614 patients admitted with SARS-CoV-2 infection to Imperial College Healthcare NHS hospitals between 25 February 2020 and 5 April 2020^24^. YLL are calculated using standard life tables^25^. Patients are individually costed using the National Cost Collection dataset from 2015 to 2019^26^ matched to HES data at Health Resource Group (HRG) level. Staff numbers are obtained from the NHS Electronic Staff Records for 2020. G&A beds are calculated using the March 2020 extract of the Quarterly Bed Availability and Occupancy Dataset^27^. CC beds are obtained from the Critical Care Monthly Situation Reports dataset for February 2020^28^. Monthly emergency admissions are obtained from the A&E Attendances and Emergency Admissions dataset from NHS England Statistics for March to June 2020^29^.

### Scenario analysis

For both OS and SP as well as under both the upper- and the lower-bound assumptions, we account for uncertainty by considering alternative scenarios based on capacity constraints, inflows of emergency admissions and pandemic trajectories. For the capacity constraints, we consider that capacity either remains at pre-pandemic levels or is expanded to reflect hospital interventions introduced to increase total capacity by 16,500 beds (for example, field hospitals) and 38,462 staff^30^ (for example, recruitment of retired and student medical staff), see Supplementary Section 3.2. For the inflows of emergency admissions, we consider that emergency admissions either remain as forecasted or that there is a reduction in the forecasted number of patients in need of emergency care (to reflect behavioral changes due to the pandemic and its mitigation strategies as well as potentially increased mortality at home). We reduce our forecasted emergency needs by 34%, using A&E attendance data to estimate the proportion of the reduction in emergency admissions throughout the pandemic (Supplementary Section 3.2). For the pandemic trajectories, and to capture a range of scenarios characterizing the severity of the pandemic and the pressure on hospital capacity, we consider the following reproduction numbers (*R*_*t*_) : *R*_*t*_ = 1.2, which yields a second-wave peak in hospital occupancy similar to the peak of the first wave; and *R*_*t*_ = 1.1, which generates a notably milder second wave of the pandemic, and a peak hospital occupancy akin to seasonal flu. These are combined with two alternative control interventions that impose lockdowns on 1 December 2020 (early LD) and 1 January 2021 (late LD).

The combination of these different assumptions leads to the following scenarios: baseline early-LD and late-LD scenarios assume an effective reproduction number of *R*_*t*_ = 1.1, pre-pandemic staff and bed capacity, and no reduction in emergencies. Best-case early- and late-LD scenarios assume *R*_*t*_ = 1.1 plus a 34% reduction in emergencies, and capacity expanded. Worst-case early- and late-LD scenarios assume *R*_*t*_ = 1.2, no reduction in emergencies and pre-pandemic capacity (Supplementary Section 8).

### Scenario results

Except where flagged, YLL are reported as the minimum (which occurs in either the best-case early-LD or the best-case late-LD scenarios) and the maximum (which occurs in either the worst-case early-LD or the worst-case late-LD scenarios) observed YLL differences across all scenarios considering both the upper- and lower-bound results. Values reported refer to the comparison of OS with SP1. Comparisons with SP2–4 are in Supplementary Fig. 6.

**Fig. 2:**
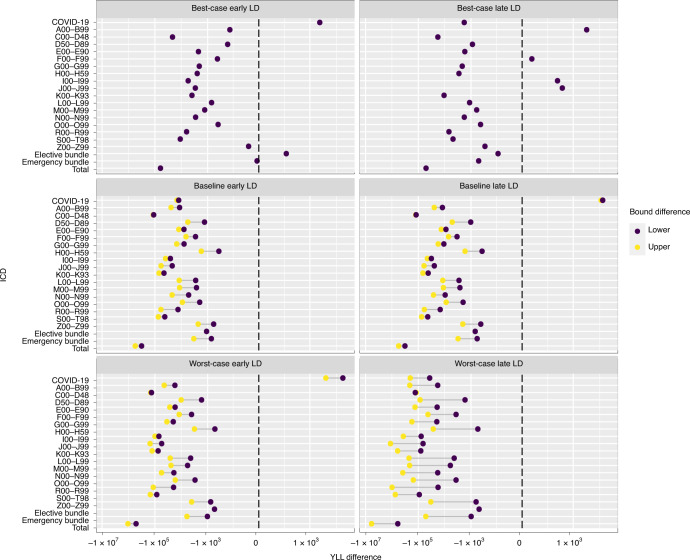
Comparison of SP1 and OS for YLL under various scenarios. Each dot represents the log of the difference in years of life lost (YLL) for all admissions under SP1 and the OS (YLL_OS_ − YLL_SP_) over the 52-week planning horizon for each ICD-10 diagnosis code. Yellow dots represent the upper-bound differences. Dark purple dots represent the lower-bound differences. The vertical black dashed lines represent no difference (of zero YLL). Supplementary Fig. 6 presents this information for SP2–4. ICD-10 codes included are A00–B99 (certain infectious and parasitic diseases), C00–D48 (neoplasms), E00–E90 (endocrine, nutritional and metabolic diseases), F00–F99 (mental and behavioral disorders), G00–G99 (diseases of the nervous system), H00–H59 (diseases of the eye and adnexa), I00–I99 (diseases of the circulatory system), J00–J99 (diseases of the respiratory system), K00–K93 (diseases of the digestive system), L00–L99 (diseases of the skin and subcutaneous tissue), M00–M99 (diseases of the musculoskeletal system and connective tissue), N00–N99 (diseases of the genitourinary system), O00–O99 (pregnancy, childbirth and the puerperium), R00–R99 (symptoms, signs and abnormal clinical and laboratory findings, not elsewhere classified), S00–T98 (injury, poisoning and certain other consequences of external causes) and V01–Y98 (external causes of morbidity and mortality). The elective bundle is composed of ICD codes A00–B99, E00–E90, F00–F99, H60–H95 (diseases of the ear and mastoid process), O00–O99, P00–P96 (certain conditions originating in the perinatal period), and Q00–Q99 (congenital malformations, deformations and chromosomal abnormalities). The emergency bundle is composed of ICD codes D50–D89 (disease of blood, blood-forming organs, and certain disorders involving the immune mechanism), H00–H59, H60–H95, P00–P96, Q00–Q99 and Z00–Z99 (factors influencing health status and contact with health services). Source data

YLL are greater under SP than OS in total (50,750–5,891,608 YLL across the scenarios) and for most disease groups and scenarios (Fig. 2). In per-capita terms and across all diseases, the largest gains accrue in the worst-case LD scenario with the OS offering up to 1.58 and 0.82 additional years of life for patients aged <25 and aged 25–64, respectively. The International Statistical Classification of Diseases and Related Health Problems (ICD) groups with the largest gains are neoplasms (C00–D48: 18,111–144,719 YLG; with the highest observed per-capita gains observed for young patients amounting to 1.1 YLG), digestive system diseases (K00–K93: 3,935–606,016 YLG; highest observed per-capita gains for young patients amounting to 1.2 YLG), and injuries and poisoning (S00–T98: 4,818–742,851 YLG; highest observed per-capita gains for young patients amounting to 1.6 YLG). Large differences are also observed for diseases of the circulatory system (I00–I99: −253–368,797 YLG; highest observed per-capita gains for young patients amounting to 2.2 YLG) and for respiratory diseases (J00–J99: −385–1,131,860 YLG; highest observed per-capita gains for young patients amounting to 1.9 YLG), see Fig. 2. OS prioritize these patients (to emergency care and to CC) over patients with COVID-19, resulting in lower YLL than SP. The highest per-capita gains of OS over SP occur in the worst-case late-LD scenario for young (<25) patients with COVID-19 (3.8 YLG) and for diseases of the circulatory system (2.2 YLG). The OS penalize the elderly patients with COVID-19 with 3.4 YLL compared with SP.

**Fig. 3:**
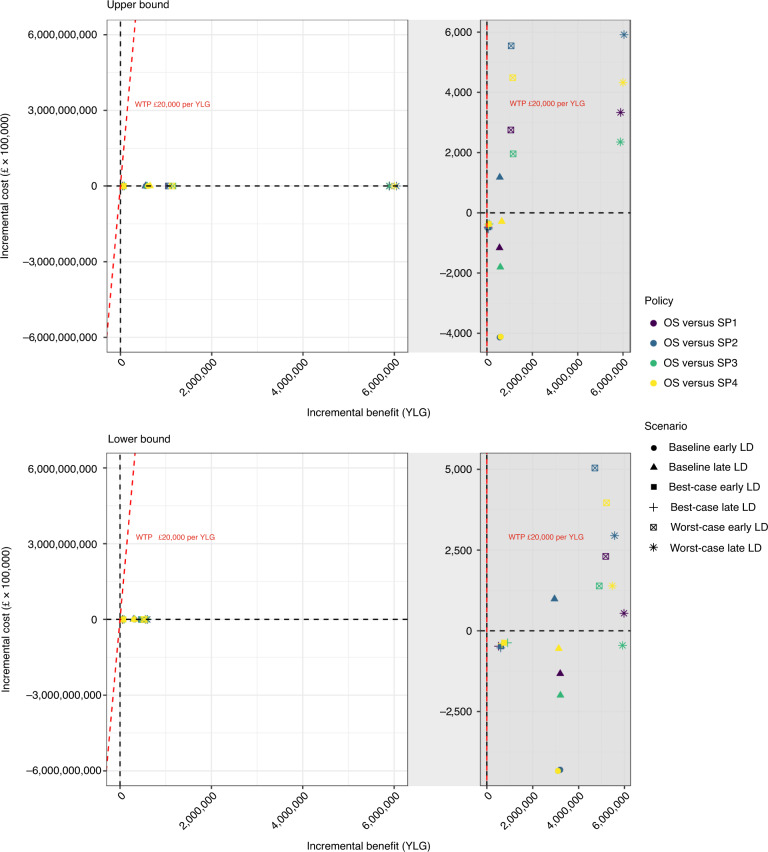
Incremental cost-effectiveness ratios/cost-effectiveness plane. Illustration of the incremental benefit (measured as years of life gained, YLG) and the incremental cost (in GBP, £) across two panels outlining incremental cost effectiveness for upper- and lower-bound estimates. These are calculated as Incremental cost = Cost_OS_ − Cost_SP_ and Incremental benefit = YLL_SP_ − YLL_OS_. The willingness-to-pay (WTP) threshold is drawn as a dashed red line with a value of £20,000 per YLG. Values below the WTP in the top-right quadrant imply that the OS are cost effective when compared with SP. Values in the bottom-right quadrant indicate that the OS are cheaper and are associated with higher YLG than the SP. Gray panels represent an enlarged view of their respective white panels. Different policy comparisons are illustrated by color: purple compares the OS with SP1, blue compares OS with SP2, green compares OS with SP3, and yellow compares OS with SP4. Different symbols represent different scenarios: dots illustrate the baseline early LD, triangles illustrate the baseline late LD, squares illustrate the best-case early LD, crosses illustrate the best-case late LD, crossed boxes illustrate the worst-case early LD and stars represent the worst-case late LD. Source data

The reduction in YLL achieved by OS is greater in scenarios where capacity constraints are more restrictive (worst-case upper bound), suggesting that an optimal prioritization of electives is increasingly beneficial as resources become scarce. When capacity enables accommodating all emergencies or there is scope to invest in extra capacity for emergencies beyond existing levels (lower bound), there is less difference between OS and SP because all patients can receive care. The notable health gains of OS do not come at increased costs in baseline and best-case scenarios (Fig. 3 and Table 1). For scenarios in which OS is costlier than SP, small extra spending is required to save many lives, with the extra costs being associated with an increased number of elective admissions and shifting from low-priority to costlier high-priority patients. In the worst-case scenarios, OS are cost effective for thresholds between £57 and £1,070 per YLL (Fig. 3 and Table 1).

**Table 1:** Health economic metrics and patient flows comparing OS versus SP1

		Health economic metrics				
				ICER calculations (OS versus SP1)		
	Scenario name	Total cost^a^ (£ millions)	Total YLL^a^ (×1,000)	Incremental YLG^a^ (×1,000)	Incremental costs^a^ (£ millions)	ICER^a^ (£ per YLG)
OS	Baseline early LD	[23,173; 23,180]	[5,113; 5,129]			
	Baseline late LD	[23,260; 23,268]	[5,350; 5,365]			
	Best-case early LD	[20,458; 20,458]	[4,313; 4,313]			
	Best-case late LD	[20,525; 20,525]	[4,548; 4,548]			
	Worst-case early LD	[23,197; 23,204]	[6,063; 6,078]			
	Worst-case late LD	[22,876; 23,151]	[7,266; 7803]			
SP1	Baseline early LD	[23,586; 23,611]	[5,432; 5,687]	[319; 559]	[−430; −413]	OS dominates
	Baseline late LD	[23,377; 23,401]	[5,669; 5,918]	[320;553]	[−133; −116]	OS dominates
	Best-case early LD	[20,506; 20,506]	[4,375; 4,375]	[61;61]	[−48; −48]	OS dominates
	Best-case late LD	[20,573; 20,573]	[4,599; 4,599]	[51; 51]	[−48; −48]	OS dominates
	Worst-case early LD	[22,922; 22,974]	[6,581; 7,130]	[518; 1,052]	[230; 275]	[445; 262]
	Worst-case late LD	[22,543; 23,097]	[7,864; 13,694]	[598; 5,892]	[54; 333]	[91; 57]

		Patient flows (×1,000)				
	Scenario name	COVID emergency admissions	Non-COVID emergency admissions	Total elective admissions	Total admission denials	
OS	Baseline early LD	179	6,554	2,205	2	
	Baseline late LD	200	6,540	2,229	2	
	Best-case early LD	182	4,294	3,868	0	
	Best-case late LD	202	4,294	3,869	0	
	Worst-case early LD	249	6,533	2,156	2	
	Worst-case late LD	311	6,487	2,021	74	
SP1	Baseline early LD	181	6,635	2,154	9	
	Baseline late LD	201	6,638	2,014	8	
	Best-case early LD	182	4,318	3,850	0	
	Best-case late LD	202	4,318	3,850	0	
	Worst-case early LD	251	6,643	1,702	19	
	Worst-case late LD	329	6,487	1,600	196	

^a^Brackets represent [lower bound; upper bound].

**Fig. 4:**
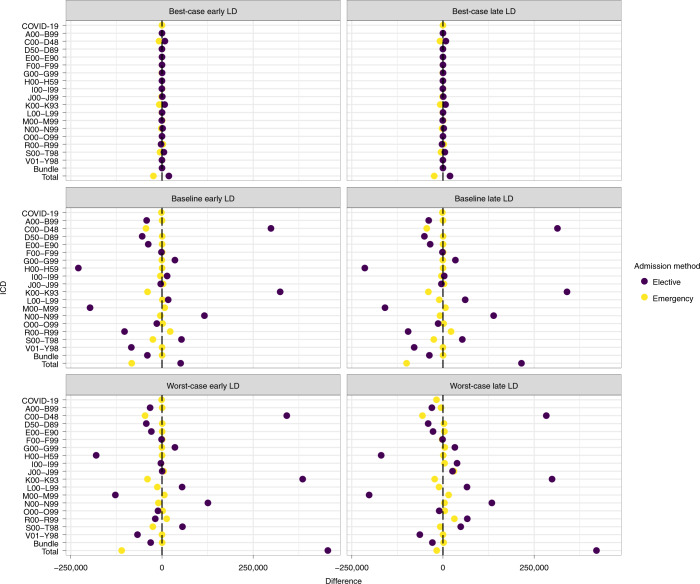
Comparison of elective and emergency admissions between OS and SP1 under various scenarios. Each dot represents the difference between the number of admissions under SP1 and OS over the 52-week planning horizon for each ICD-10 diagnosis code. This is calculated as Difference = Admissions_OS_ − Admissions_SP_. The vertical black dashed lines represent no difference. Dark purple dots represent elective admissions. Yellow dots represent emergency admissions. Supplementary Fig. 7 presents this information for SP2–4. ICD-10 codes included are A00–B99 (certain infectious and parasitic diseases), C00–D48 (neoplasms), E00–E90 (endocrine, nutritional and metabolic diseases), F00–F99 (mental and behavioral disorders), G00–G99 (diseases of the nervous system), H00–H59 (diseases of the eye and adnexa), I00–I99 (diseases of the circulatory system), J00–J99 (diseases of the respiratory system), K00–K93 (diseases of the digestive system), L00–L99 (diseases of the skin and subcutaneous tissue), M00–M99 (diseases of the musculoskeletal system and connective tissue), N00–N99 (diseases of the genitourinary system), O00–O99 (pregnancy, childbirth and the puerperium), R00–R99 (symptoms, signs and abnormal clinical and laboratory findings, not elsewhere classified), S00–T98 (injury, poisoning and certain other consequences of external causes) and V01–Y98 (external causes of morbidity and mortality). The elective bundle is composed of ICD codes A00–B99, E00–E90, F00–F99, H60–H95 (diseases of the ear and mastoid process), O00–O99, P00–P96 and Q00–Q99 (congenital malformations, deformations and chromosomal abnormalities). The emergency bundle is composed of ICD codes D50–D89 (disease of blood, blood-forming organs, and certain disorders involving the immune mechanism), H00–H59, H60–H95, P00–P96, Q00–Q99 and Z00–Z99 (factors influencing health status and contact with health services). Source data

OS accommodate more elective admissions than SP since there is no blanket postponement of elective procedures, and admission scheduling is determined by the patient’s probability of survival and likelihood of needing emergency care while waiting for elective care. When capacity constraints are less restrictive (baseline and best-case early LD/late LD, worst-case early LD), OS exhibit fewer non-COVID-19 patients requiring emergency care than SP (Table 1). The ICDs for which the difference in elective (emergency) admissions is the highest (lowest) are digestive system diseases (K00–K93), neoplasms (C00–D48), genitourinary system disease (N00–N99), and injuries and poisoning (S00–T98) (Fig. 4 for SP1 and Supplementary Fig. 7 for SP2–4).

In the worst-case scenarios, capacity constraints are more stringent than in the baseline and best-case scenarios, and more patients require emergency care while waiting for elective care under both OS and SP. Due to resource constraints, differences in emergency admissions between OS and SP are smaller in the worst-case late-LD scenario (Table 1). Notice that there are no denials in emergency admissions in the best-case scenarios for either model. However, this is not the case in any other scenario, where emergency patients are denied admission due to capacity constraints. The number of emergency admission denials is higher for SP than OS (Table 1).

**Fig. 5:**
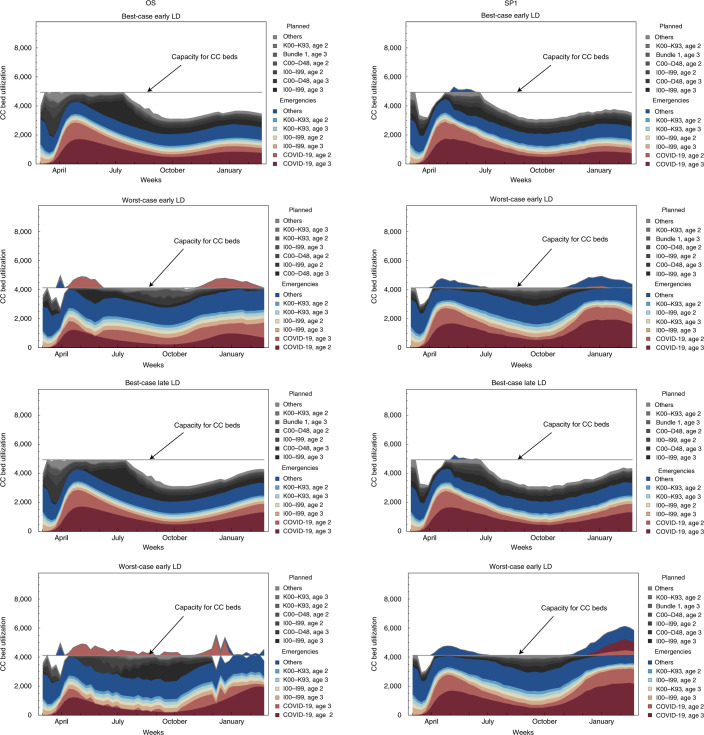
CC bed utilization by patient group for the OS and SP1 over the 52-week planning horizon. Comparison of bed utilization for the OS (left column) and SP1 (right column) under four scenarios (each row represents one scenario) over the 52-week planning horizon. Each subplot reports, using ICD-10 diagnosis code, the six patient groups with the highest bed utilization rates for both emergency (with red–blue tones) and planned (with gray tones) procedures. Emergency patient groups that cannot be accommodated in the critical care units are reported above the horizontal critical care capacity line. ICD codes included are C00–D48 (neoplasms), I00–I99 (diseases of the circulatory system) and K00–K93 (diseases of the digestive system). Bundle 1 includes ICD codes A00–B99 (certain infectious and parasitic diseases), E00–E90 (endocrine, nutritional and metabolic diseases), F00–F99 (mental and behavioral disorders), H60–H95 (diseases of the ear and mastoid process), O00–O99 (pregnancy, childbirth and the puerperium), P00–P96 (certain conditions originating in the perinatal period) and Q00–Q99 (congenital malformations, deformations and chromosomal abnormalities). Ages 1, 2 and 3 correspond to age brackets <25, 25–64 and 64+, respectively. Source data

At baseline, OS deny CC to few patients (2.2% and 2.6% over the 52 weeks under early LD and late LD, respectively), most of which (83% and 85%) are patients with COVID-19 aged 65+ (Supplementary Fig. 9). In the worst-case scenarios, the share of patients denied CC increases to 4.8% and 6.4% under early LD and late LD, with 92% and 83% of patients denied CC stemming from COVID-19 aged 65+ (Fig. 5). Therefore, OS show that YLL are minimized when non-COVID-19 patients are prioritized for CC over patients with COVID-19. In the worst-case late-LD OS, patients with neoplasms and diseases of the circulatory system are prioritized over patients with COVID-19 aged 65+ (see Supplementary Fig. 10 for G&A bed utilization). In the best-case scenarios, OS admit to CC all patients requiring it, while SP deny CC to patients during the weeks following the cancellation of elective procedures (Table 1).

## Discussion

In England, fears over premature mortality and morbidity associated with the postponement of elective procedures prompted policy discussions on how to accommodate patients needing elective care^4^. NHS England directed hospitals to sustain elective care over the winter 2020–2021. This directive is proving difficult to meet with demands from patients with COVID-19 surging^31,32^. Our findings are timely for the NHS as they have the potential to support re-scheduling delayed elective procedures while coping with further increases in patients with COVID-19.

While there is uncertainty about our estimates, reflected in the variability of the orders of magnitude of the reported gains of the OS over SP across the different scenarios, the scenarios and respective parameters reflect a broad spectrum of stringency of capacity constraints. As such, gains of OS over SP are characterized across plausible states of the world. Importantly, our results highlight some high-level commonalities across all scenarios that are useful to inform the formulation of national guidance akin to the guidance developed by the National Institute for Excellence Health and Care Excellence (NICE). Our findings outline life-saving key prioritization principles that can be embedded in national policies: (1) consider the relative capacity to benefit of patients with different diseases and ages; (2) postpone elective treatments for which disease progression is mild and that have lower chances of being admitted to emergency care; (3) prioritize access to emergency care and critical care based on capacity to benefit, rather than by default prioritizing one disease (for example, COVID-19) over other patients (for example, non-COVID-19)^2^. Where data are not available, these general principles can be used by policymakers globally to rapidly formulate national guidance in the future when national health services experience severe demand shocks such as those experienced during the pandemic. Our model is open source, and it is solved within minutes on a standard computer^33,34^.

While our objective is to inform the development of national guidance analogous to NICE guidance, our model does not capture variations between health authorities in provision or efficiency of implementation of the prioritization rules. However, the model can be applied to smaller geographical units that can be of use in decentralized health systems (for example, Italy or Canada). With regards to single hospitals capacity planning, while national guidance could in principle be mandatory, we would argue that—in the case of our modeling—localities should have autonomy to adapt and refine recommendations depending on the opportunities and constraints offered by local service delivery systems and population needs if, for example, they have excess capacity for certain treatments that would otherwise remain unused.

On the other hand, we do not envisage our model being used at a hospital level, as this would require detailed modeling of hospital-level capacity (by treatment) and patient-level constraints, which in turn would require discrete decision variables that would lead to much harder optimization problems.

This study has several limitations. The impact of COVID-19 on staff shortages and infection-control measures (for example, ward closures) are not modeled, which probably underestimates its impact on hospital capacity. Referrals for elective care are assumed to remain constant at pre-pandemic levels, but it is likely that primary care attendance and referrals were reduced during the pandemic^35^. This has two implications for the analysis: (1) we do not account for YLL due to reduced care-seeking behavior by patients; (2) the types of referral may differ because of the pandemic, thus impacting costs and YLL of policies.

Patients within each group are considered homogenous in clinical severity and disease progression, and we do not consider competing risks that could change prioritization rules. Also, patients scheduled for elective care may be heterogeneous on disease progression and probability of dying. Without an indicator of severity valid across ICDs, we account for latent severity by modeling transition probabilities as a function of waiting times and through some patients being forecasted to need care earlier than others based on historical data. Thus, this analysis may underestimate costs and YLL.

Some patients who have been scheduled for elective care may subsequently die of a hospital-acquired COVID-19 infection; this is not accounted for in our study. If granular data were available, this could be easily accounted for in the modeling of the transition probabilities. At present, the significance of hospital-acquired infections in the context of our findings is unclear. On the one hand, one could argue that due to capacity shortages, hospitals would use elective wards to admit more emergency patients with COVID-19, thus possibly increasing the risk of hospital-acquired infections. On the other hand, many patients with COVID-19 in the United Kingdom have been allocated to dedicated hospitals (for example, the Nightingale and field hospitals), and the infection-control programs and protocols developed during the pandemic have helped to reduce hospital COVID-19 transmissions^36^, thus mitigating the risk of nosocomial COVID-19 in hospitalized patients.

We do not consider treatment preferences of patients, the public or medical professionals^37^. While our model largely disregards the impact on health inequalities, it embraces the equity principle that a life year gained is of equal value, regardless of who receives it, which is akin to what is considered by NICE for priority setting. Socioeconomic deprivation and minority ethnicity is associated with increased COVID-19 mortality risk^38,39^. Further research is needed to ensure that OS do not inadvertently increase health inequalities, and are acceptable to clinicians, patients and the public. If data are available, the model can incorporate health inequalities.

Our objective function minimizes YLL because that is aligned with many prioritization policies routinely used by the NHS in England. For example, the underlying assumption in NICE guidance, followed also internationally, is that the limited NHS budget should be used to maximize health outcomes, albeit using quality adjusted life years (QALYs) rather than YLL. Although QALYs are used by many health systems for economic evaluations of health treatments, data are not routinely available for all diseases and health states for patients in hospital care settings. If data were available, it would in principle be possible to adapt the model to any other objective function, including the maximization of QALYs as well as any other equity considerations.

While these are important caveats that can impact the estimated YLL and costs, they are likely to affect OS and SP in a similar way, thus not impacting their comparison.

The presented model shows that changes in prioritization rules can minimize the detrimental health impact of unprecedented hospital capacity shortages during the pandemic. It operationalizes the principles of best use of limited resources underlying the management of the English health system^40,41^. More generally, the model is of relevance to health systems globally seeking to prioritize hospital care, substantially improving on short-sighted measures that focus on patients with COVID-19 to the detriment of the health of other patients.

## Methods

### Optimized schedules

OS are obtained from the solution of an LP model. In the following, we present an abridged model that contains the key decision variables, constraints and input parameters (cf. Supplementary Table 2) of the LP formulation. A more detailed description of the entire optimization model is relegated to Supplementary Section 1.1.

Our LP model can be interpreted as a multiperiod inventory model with five classes of non-negative decision variables: (1) *w*, tracking patients waiting for care; (2) *z*, recording which patients are admitted to hospital in a particular week; (3) *z*′, tracking the initial state of admitted patients (G&A, CC or death because of denied care due to capacity shortages); (4) *y*, tracking the state of hospitalized patients; (5) *x*, mapping the transitions of patients across states $s,s^\prime \in {{{\mathcal{S}}}} = \left\{ {G,C,G^ \ast ,H,D} \right\}$, where *G*, *C* and G* correspond to patients in G&A, CC and G&A after having been denied CC, respectively, while *H* and *D* record the recovered and deceased patients.

The abridged LP formulation reads as follows.

1 $$\min \;{\mathrm{YLL}} = \mathop {\sum }\limits_{t \in {{{\mathcal{T}}}}} \mathop {\sum }\limits_{p \in {{{\mathcal{P}}}}} \mathop {\sum }\limits_{a \in {{{\mathcal{A}}}}} \lambda _p\left( {y_{tpa}^D + z_{tpa}^{{\prime} D}} \right)$$

2 $${{{\mathrm{s}}}}{{{\mathrm{.t}}}}{{{\mathrm{.}}}}\;w_{t + 1,p} = \phi _{tp}^n + \left( {1 - \pi _{w,p}^e} \right)w_{tp} - z_{tpn}\quad \forall t \ne T,\forall p$$

3 $$z_{tpe} + z_{tpe}^{{\prime}D} = \phi _{tp}^e + \pi _{w,p}^ew_{tp}\quad \forall t \ne T,\forall p$$

4 $$z_{tpa}^{{\prime}s} = z_{tpa}\pi _{z,tpa}^s\quad \forall t \ne T,\forall p,\forall s \in \left\{ {G,C} \right\},\forall a$$

5 $$y_{t + 1,pa}^s = \mathop {\sum }\limits_{s^\prime \notin \left\{ {H,D} \right\}} x_{tpa}^{s^{\prime} s}\quad \forall t \ne T,\forall p,\forall s,\forall a$$

6 $$x_{tpa}^{s^{\prime} s} = \pi _{y,pa}^{s^{\prime} s}\left( {y_{tpa}^{s^\prime } + {z^{\prime}} _{tpa}^{s^\prime }} \right)\quad \forall t \ne T,\forall p,\forall s^\prime \in \left\{ {G,C,G^ \ast } \right\},\forall s \in \left\{ {G,H,D} \right\},\forall a$$

7 $$x_{tpa}^{sC} + x_{tpa}^{sG^ \ast } = \pi _{y,pa}^{sC}\left( {y_{tpa}^s + z_{tpa}^{{\prime}s}} \right)\quad \forall t \ne T,\forall p,\forall s \in \left\{ {G,C,G^ \ast } \right\},\forall a$$

The model minimizes the total YLL over a 52-week planning horizon $t \in {{{\mathcal{T}}}} = \left\{ {1, \ldots ,52} \right\}$ across all patient groups $p \in {{{\mathcal{P}}}}$, each of which comprises patients of a particular disease type, age group and admission type $a \in {{{\mathcal{A}}}} = \left\{ {n,e} \right\}$, where *n* and *e* refer to elective and emergency admissions, respectively. The YLL is calculated as the specific YLL per patient group $\lambda _p$ multiplied by the number of deaths during care ($y_{tpa}^D$) and due to denial of care ($z_{tpa}^{{\prime} D}$) (equation (1)). Other objective functions, such as the minimization of the healthcare costs, or combinations of YLL and healthcare costs, can be readily employed by modifying equation (1) accordingly.

At the beginning of week *t*, an exogenous inflow of patients in need of care ($\phi _{tp}^a$) is observed for every patient group $p \in {{{\mathcal{P}}}}$ and admission type $a \in {{{\mathcal{A}}}}$. Some of the elective patients are admitted to hospital during week *t* ($z_{tpn}$), while the other elective patients remain in the waiting queue or require emergency care (with probability $\pi _{w,p}^e$) (equation (2)). Incoming emergency patients ($\phi _{tp}^e$) are always admitted if capacity is available. In case of capacity shortages, admission to hospital might be denied to patients in need of emergency care; in the LP model, we conservatively assume that these patients ($z_{tpe}^{{\prime} D}$) die due to the lack of the required emergency treatments (equation (3)) (this assumption is relaxed in the sensitivity analysis).

When admitted to hospital, the fraction $\pi _{z,tpa}^G$ ($\pi _{z,tpa}^C$) of patients requiring a G&A (CC) bed is recorded by the decision variables $z_{tpa}^{{\prime}G}$ ($z_{tpa}^{{\prime}C}$) (equation (4)). The number of patients of patient group $p \in {{{\mathcal{P}}}}$ and admission type $a \in {{{\mathcal{A}}}}$ in a given state $s \in {{{\mathcal{S}}}}$ at the end of week *t*, $y_{t + 1,pa}^s$, is equal to the sum of the patients who remained in state *s* during that week ($x_{tpa}^{ss}$) and the transitions from other states $s^\prime \in {{{\mathcal{S}}}}$ to *s* during week *t* ($x_{tpa}^{s^\prime s}$) (equation (5)). Transitions across states evolve according to the transition probabilities $\pi _{y,pa}^{s^\prime s}$, and they are recorded by the decision variables $x_{tpa}^{s^\prime s}$ (equation (6)). Admission to CC is an exception to this: equation (7) enforces that in case of capacity shortages, CC might be denied to a patient in need; the patient then transitions to a designated G&A state (G*) where s/he evolves according to a new set of transition probabilities until CC capacity becomes available.

The abridged model is a simplified model of the OS and does not account for the possibility to send patients requiring CC to G&A (the designated state G*) directly upon admission, it assumes that all admitted patients are hospitalized for at least one week, and it disregards the resource constraints of the health system. Those considerations are accounted for by our OS, and they are presented in Supplementary Section 1.1, which also contains more detailed explanations. The solution time of the LP model scales polynomially in the size of the input data, that is, the number of time periods, the number of patient groups as well as the number of resources. Note in particular that the solution time is unaffected by the number of patients, which makes our model particularly suited to policy advice at a national level.

### Model inputs

Our model relies on weekly projections of key estimates over the 52-week horizon: patients requiring elective care (Supplementary Section 3.1), non-COVID-19 patients and patients with COVID-19 requiring emergency care (Supplementary Sections 3.1 and 4); probabilities of transitioning to various states once admitted (for example, discharged, to CC, to G&A, died) (Supplementary Section 5.2), and probabilities of patients waiting for elective care needing emergency care (Supplementary Section 5.1). Additional inputs are: forecasts of the number of patients waiting for elective care; hospitalized patients in week 0; costs and YLL of all patients (Supplementary Sections 6.1 and 6.2). Capacity constraints on the supply side are given by the maximum number of G&A/CC beds and staff (senior and junior doctors, and nurses) and recommended staff-to-bed ratios^42,43^. To reflect historical bed utilization rates, we assume all available capacity can be used.

Patients in need of care are categorized into groups based on primary diagnosis and age resulting in 42 and 45 disease-age groups for non-COVID-19 elective and emergency admissions, respectively, and 3 groups for COVID-19 emergency admissions (Supplementary Section 2). A binary frailty score is calculated for each patient (Supplementary Section 3)^44^.

Non-COVID-19 patients are forecasted using time-series methods assuming historical hospital bed utilization rates (Supplementary Section 3.1). Patients with COVID-19 are projected with a SEIR model using compartments for three age groups and degrees of severity, hospitalizations and deaths. The basic reproduction number, seed time of the epidemic, start of lockdown and reduction in transmission due to non-pharmaceutical interventions are calibrated to hospital occupancy data (Supplementary Section 4)^45^.

The disease progression of patients waiting for elective care may be such that they transition to the cohort requiring emergency care with a probability estimated as a function of waiting time (days) using a Kaplan–Meier estimator (Supplementary Section 5.1). Once patients are admitted for either elective or emergency care to either G&A or CC, they can transition to any of the following states: (1) remain in their current state (G&A or CC); (2) move to CC or G&A; (3) be discharged alive; or (4) die. The transition probabilities are estimated using multinomial logistic regressions, conditional on waiting time for electives (Supplementary Section 5.2).

We compute mean treatment costs for each patient group by averaging across the individual average cost of every non-COVID-19 patient in English hospitals. As the cost of patients with COVID-19 is yet unknown, we determine their HRGs in three London hospitals using the 2015–2019 national cost schedule (Supplementary Section 6.1). We calculate aggregate YLL by adding the YLL for all deaths within each age group, averaging age specific remaining life expectancies across all ages within the group (Supplementary Section 6)^25^.

### Ethical approval

The study was approved by the NHS Digital Independent Group Advising on the Release of Data (IGARD) committee (application reference: DARS-NIC-276970). No other ethical approval was required.

### Supplementary information

Supplementary Information Supplementary Sections 1–10, Figs. 1–10 and Tables 1–11.

Supplementary Data 1 Source data used for Supplementary Fig. 3.

Supplementary Data 2 Source data used for Supplementary Fig. 4.

Supplementary Data 3 Source data used for Supplementary Fig. 5.

Supplementary Data 4 Source data used for Supplementary Fig. 6.

Supplementary Data 5 Source data used for Supplementary Fig. 7.

Supplementary Data 6 Source data used for Supplementary Fig. 8.

Supplementary Data 7 Source data used for Supplementary Fig. 9.

Supplementary Data 8 Source data used for Supplementary Fig. 10.

### Source data

Source Data Fig. 2 Statistical source data for Fig. 2.

Source Data Fig. 3 Statistical source data for Fig. 3.

Source Data Fig. 4 Statistical source data for Fig. 4.

Source Data Fig. 5 Modeling results, reported in eight different sheets corresponding to the eight different subplots in Fig. 5.

## Data Availability

Due to the data sharing agreement, data cannot be made publicly available with this study. Individual-level data used for the analysis of COVID-19 outcomes are not publicly available and only accessible to selected researchers. Data from Hospital Episode Statistics and Electronic Staff Records are available to all researchers upon making an application to NHS Digital. Applications can be made through the NHS’s Data Access Request Service (https://digital.nhs.uk/services/data-access-request-service-dars). A&E Attendances and Emergency Admissions data are freely available at https://www.england.nhs.uk/statistics/statistical-work-areas/ae-waiting-times-and-activity/. Bed Availability and Occupancy data are freely available at https://www.england.nhs.uk/statistics/statistical-work-areas/bed-availability-and-occupancy/. Critical Care Bed Capacity data are freely available at https://www.england.nhs.uk/statistics/statistical-work-areas/critical-care-capacity/. NHS Cost Collection data are freely available at https://www.england.nhs.uk/national-cost-collection/. All model input data are documented in the Supplementary Information and made available open source at https://github.com/HFAnalyticsLab/overflow_analysis. Source Data for Figs. 2–5 and for Supplementary Figs. 3–10 are provided with this paper.

## Code availability

The source code for the optimization and simulation models used in this study is available open source^33,34,46^.
