# Supplementary material for: Optimal national prioritization policies for hospital care during the SARS-CoV-2 pandemic
Source: Nat Comput Sci. 2021 Aug 13;1(8):521–31. doi: 10.1038/s43588-021-00111-1 (PMC10766519; doi:10.1038/s43588-021-00111-1)
Supplement: Supplementary file 1 — Supplementary Sections 1–10, Figs. 1–10 and Tables 1–11. [file 43588_2021_111_MOESM1_ESM.pdf]

nature portfolio

https://doi.org/10.1038/s43588-021-00111-1

# Supplementary information

# Optimal national prioritization policies for hospital care during the SARS-CoV-2 pandemic

In the format provided by the authors and unedited

## Supplementary Information

## Supplementary Section 1: Optimization Model

### 1.1. Overview of the Model Structure

The Optimized Schedules (OS) are based on a linear programming (LP) model that optimally schedules the admission of patients to hospital under different pandemic scenarios. Further mathematical justification for our LP model can be found in the accompanying paper (D'Aeth et al. 2021).[1]

**Model inputs.** Focusing on the entire NHS in England, we first characterize (i) the initial situation (at $t = 0$) in terms of the available resources ($\Xi$) and the current allocation of patients (waiting vs in-hospital patients, in critical care (CC) vs General and acute care (G&A), etc.). We divide patients into different patient groups (as detailed in Supplementary Section 2) and subdivide each group on the basis of severity. For each subgroup, we provide as inputs (ii) their resource requirements ($\Delta$) as well as transition matrices ($\Pi$) representing the probabilities of endogenous transfers of patients between severity groups (e.g., patients needing emergency care while waiting for elective care, or patients in G&A requiring CC). For $t > 0$, based on the scenarios we are investigating (e.g. lockdown), we observe (iii) new exogenous inflows of patients ($\Phi$). Investments to increase capacity could additionally be accounted for in a strategic planning problem.

**Model outputs.** During each week, the model optimizes the allocation of patients, that is, how many patients of each group to admit to hospital ($z_t, z_t'$) as well as the in-hospital transfers of patients ($x_t, x_t'$). Crucially, we account for the possibility of capacity shortages, which, for instance, have affected patients' welfare negatively during the first peak of the COVID-19 outbreak; that is, the model considers that admission to hospital or to CC might be denied to patients in need. The objective is to minimize the total YLL (the model could also be used to minimize total cost). The key constraints are the capacities and resource balances.

### 1.2. LP Model Formulation

In this section we detail the sets, parameters, decision variables (Supplementary Tables 1, 2, and 3, respectively) and constraints of the LP optimization model. Supplementary Figures 1 and 2 offer schematic representations of the system's evolution for any given week $t$. Week $t$ begins at time $t$ and ends at time $t + 1$. Patient inflows are observed at the middle of each week (time $t + 0.5$), when also decisions on hospital admissions are made ($z_t$). The evolution of newly admitted patients during their first 3.5 days in hospital is mapped by the decision variables $x_t'$. During the following weeks, the transition of patients across severity states is mapped by the decision variables $x_t$ (see Supplementary Figure 2). The number of waiting ($w_t$) and hospitalized ($y_t$) patients is assessed at each time instant $t \in \{0, \dots, t, \dots, T\}$. The model is initialized with the number of waiting and hospitalized patients at the beginning of the planning horizon $t = 0$ ($w_0, y_0$).

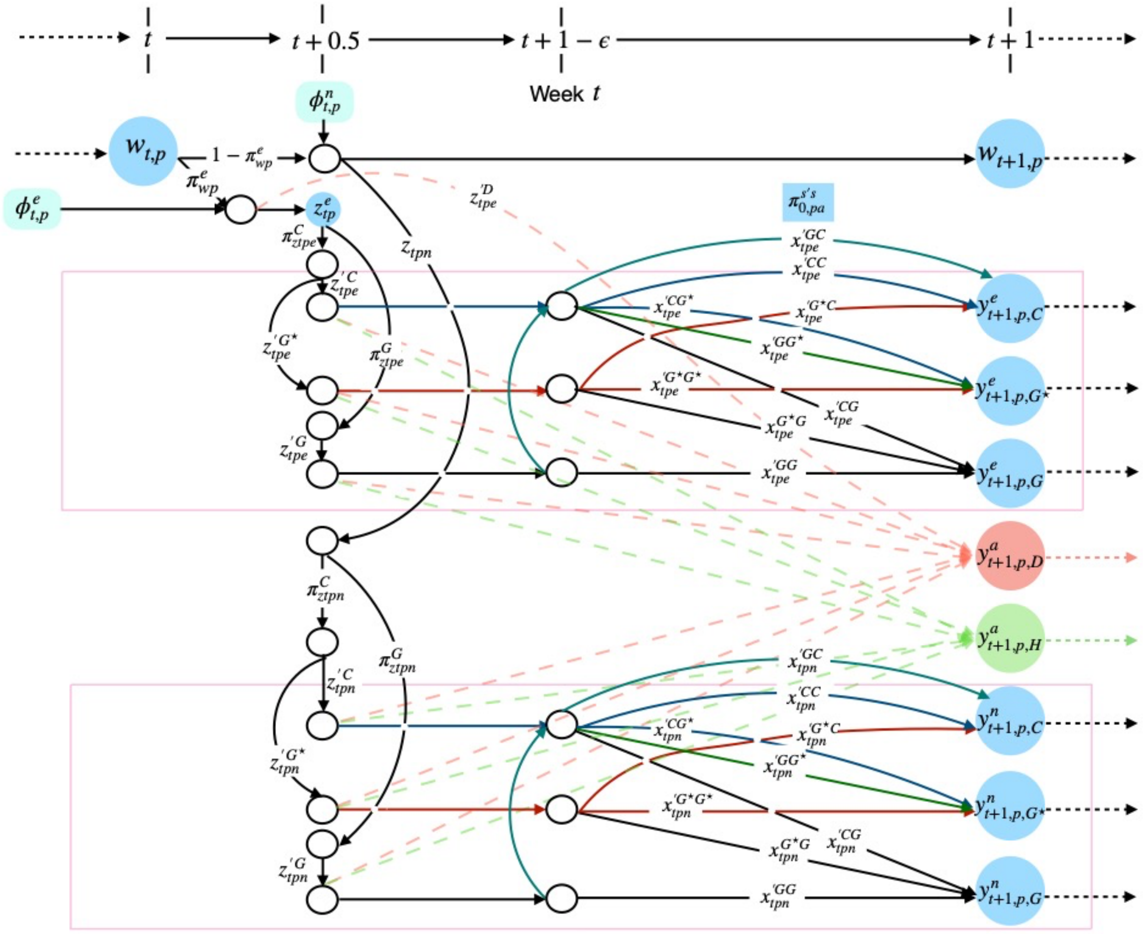


Supplementary Figure 1. Schematic overview of the system evolution of incoming patients mid-week for any given week t

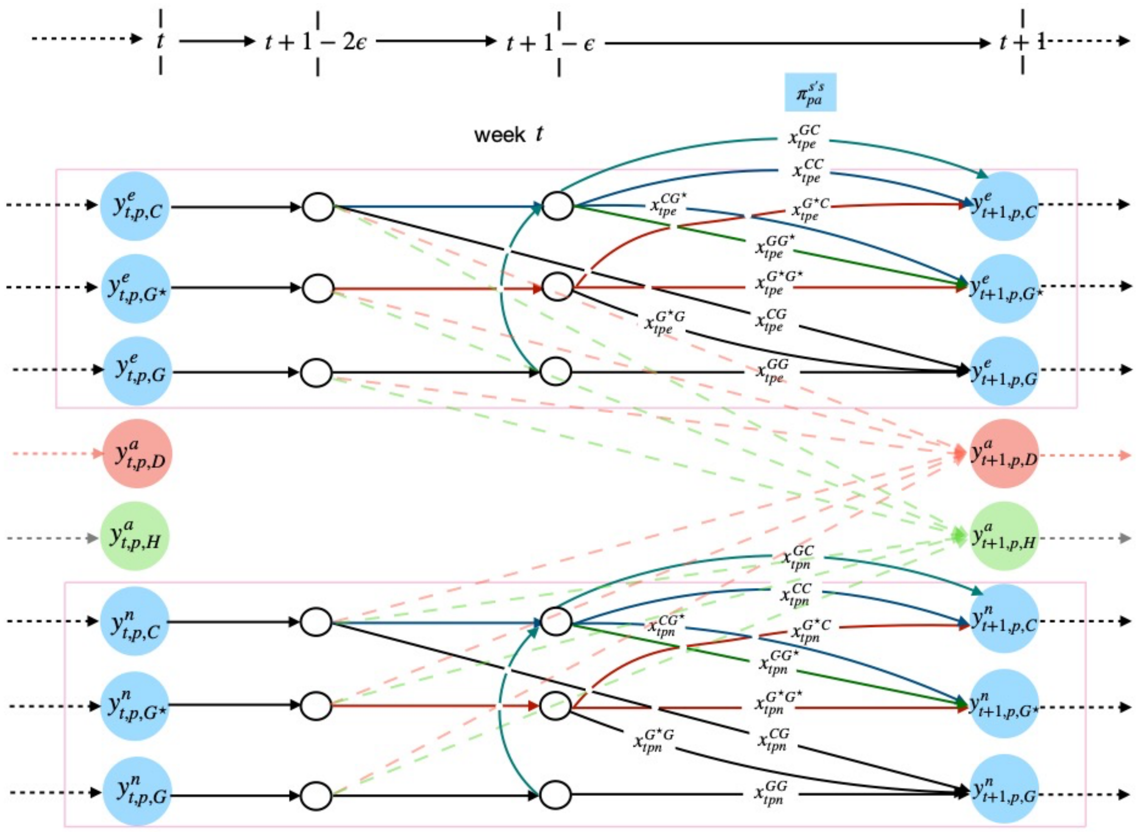


Supplementary Figure 2. Schematic representation of the system evolution of hospitalized patients during any given week t.

Supplementary Table 1. Set names and elements

| Set name (index) | Elements |
|---|---|
| TIMES ($\mathcal{T}$) | $\{0, \dots, t, \dots, T = 52\}$ Time periods (weeks) |
| RESOURCES ($\mathcal{R}$) | $\{0, \dots, r, \dots, R\}$ Resources (CC beds, G&A beds, staff) |
| PATIENT GROUPS ($\mathcal{P}$) | $\{0, \dots, p, \dots, P\}$ Patients divided by disease type and age group |
| ADMISSION TYPES ($\mathcal{A}$) | $\{e, n\}$, where $e$ is emergency and $n$ is elective admission |
| SEVERITY STATES ($\mathcal{S}$) | $\{G, C, G^*, H, D\}$, where $G$ is G&A, $C$ is CC and $G^*$ is G&A for patients who have been denied CC, respectively, while $H$ is recovered and $D$ is dead |

Supplementary Table 2. Parameters with description

| Parameter | Units | Description |
|---|---|---|
| $\phi_{tp}^{a}$ | [# patients] | New patients inflow (exogenous) for each patient group $p \in \mathcal{P}$ of admission type $a \in \mathcal{A}$ during each week $t \in \mathcal{T}$ |
| $\pi_{w,p}^{e}$ | [-] | Probability of transfer from elective $n$ to emergency $e$ for each waiting patient group $p \in \mathcal{P}$ |
| $\pi_{z,tpa}^{s}$ | [-] | Fraction of patients from each patient group $p \in \mathcal{P}$ of type $a \in \mathcal{A}$ requiring admission to $s \in \mathcal{S}$ at the beginning of each week $t \in \mathcal{T}$ |
| $\pi_{0,pa}^{ss'}$ | [-] | Probability of transfer in the first 3.5 days from severity state $s \in \mathcal{S}$ to $s' \in \mathcal{S}$ for each patient group $p \in \mathcal{P}$ of admission type $a \in \mathcal{A}$ |
| $\pi_{y,pa}^{ss'}$ | [-] | Probability of transfer (weekly transitions) from severity state $s \in \mathcal{S}$ to $s' \in \mathcal{S}$ for each patient group $p \in \mathcal{P}$ of admission type $a \in \mathcal{A}$ |
| $\delta_{0,psa}^{r}$ | [# items] | Requirement of resource $r \in \mathcal{R}$ for each patient group $p \in \mathcal{P}$ in severity state $s \in \mathcal{S}$ and admission type $a \in \mathcal{A}$ (first 3.5 days) |
| $\delta_{s}^{r}$ | [# items] | Requirement of resource $r \in \mathcal{R}$ for patients in severity state $s \in \mathcal{S}$ (weekly) |
| $\xi_{r}$ | [# items] | Capacity of resource $r \in \mathcal{R}$ (weekly) |
| $\lambda_{p}$ | [# years] | Years of life lost (YLL) for each patient group $p \in \mathcal{P}$ |
| $\gamma_{pa}$ | [GBP] | Unit cost of care for each patient group $p \in \mathcal{P}$ of admission type $a \in \mathcal{A}$ |

Supplementary Table 3. Decision variables with description

| Variable | Units | Description |
|---|---|---|
| $w_{tp}$ [(1)] | [# patients] | Elective patients of group $p \in \mathcal{P}$ *waiting* for care at time $t \in \mathcal{T}$ |
| $z_{tpa}$ | [# patients] | Patients of group $p \in \mathcal{P}$ and admission type $a \in \mathcal{A}$ *admitted* to hospital in week $t \in \mathcal{T}$ |
| $z'^{s}_{tpa}$ | [# patients] | Patients of group $p \in \mathcal{P}$ and admission type $a \in \mathcal{A}$ *admitted* in severity state $s \in \mathcal{S}$ in week $t \in \mathcal{T}$ |
| $y^{s}_{tpa}$ [(1)] | [# patients] | Patients of group $p \in \mathcal{P}$ and admission type $a \in \mathcal{A}$ *in* hospital and *in* severity state $s \in \mathcal{S}$ at time $t \in \mathcal{T}$ |
| $x^{ss'}_{tpa}$ | [# patients] | Patients of type $p \in \mathcal{P}$ of admission type $a \in \mathcal{A}$ *transferred from* severity state $s \in \mathcal{S}$ *to* $s' \in \mathcal{S}$ during week $t \in \mathcal{T}$ (weekly transitions) |
| $x'^{ss'}_{tpa}$ | [# patients] | Patients of type $p \in \mathcal{P}$ of admission type $a \in \mathcal{A}$ *transferred from* severity state $s \in \mathcal{S}$ *to* $s' \in \mathcal{S}$ during week $t \in \mathcal{T}$ (first 3.5 days) |

(1) For $t = 0$, $w_{0p}$ and $y^{S}_{0pa}$ are input parameters

Note: All variables are continuous and non-negative unless otherwise indicated

### 1.3 Constraints

The model is expressed by the objective function [1] and the constraints [2]-[15].

$$\min \quad YLL = \sum_{t\in\mathcal{T}}\sum_{p\in\mathcal{P}}\sum_{a\in\mathcal{A}} \lambda_p \left(y^D_{tpa} + z'^D_{tpa}\right) \quad [1]$$

$$\text{s.t.} \quad w_{t+1,p} = \phi^n_{tp} + \left(1 - \pi^e_{w,p}\right) w_{tp} - z_{tpn} \qquad \forall t \neq T, \forall p \quad [2]$$

$$z_{tpe} + z'^D_{tpe} = \phi^e_{tp} + \pi^e_{w,p} w_{tp} \qquad \forall t \neq T, \forall p \quad [3]$$

$$z'^C_{tpa} + z'^{G^*}_{tpa} = z_{tpa}\pi^C_{z,tpa} \qquad \forall t \neq T, \forall p, \forall a \quad [4]$$

$$z'^G_{tpa} = z_{tpa}\pi^G_{z,tpa} \qquad \forall t \neq T, \forall p, \forall a \quad [5]$$

$$y^s_{t+1,pa} = \sum_{s' \notin \{H,D\}} \left(x^{s's}_{tpa} + x'^{s's}_{tpa}\right) \qquad \forall t \neq T, \forall p, \forall s, \forall a \quad [6]$$

$$x'^{s's}_{tpa} = \pi^{s's}_{0,pa} z'^{s'}_{tpa} \qquad \forall t \neq T, \forall p, \forall s' \in \{G, C, G^*\}, \forall s \in \{G, H, D\}, \forall a \quad [7]$$

$$x'^{CC}_{tpa} + x'^{CG^*}_{tpa} = \pi^{CC}_{0,pa} z'^C_{tpa} \qquad \forall t \neq T, \forall p, \forall a \quad [8]$$

$$x'^{GC}_{tpa} + x'^{GG^*}_{tpa} = \pi^{GC}_{0,pa} z'^G_{tpa} \qquad \forall t \neq T, \forall p, \forall a \quad [9]$$

$$x'^{G^*C}_{tpa} + x'^{G^*G^*}_{tpa} = \pi^{G^*C}_{0,pa} z'^{G^*}_{tpa} \qquad \forall t \neq T, \forall p, \forall a \quad [10]$$

$$x^{s's}_{tpa} = \pi^{s's}_{y,pa} y^{s'}_{tpa} \qquad \forall t \neq T, \forall p, \forall s' \in \{G, C, G^*\}, \forall s \in \{G, H, D\}, \forall a \quad [11]$$

$$x^{CC}_{tpa} + x^{CG^*}_{tpa} = \pi^{CC}_{y,pa} y^C_{tpa} \qquad \forall t \neq T, \forall p, \forall a \quad [12]$$

$$x^{GC}_{tpa} + x^{GG^*}_{tpa} = \pi^{GC}_{y,pa} y^G_{tpa} \qquad \forall t \neq T, \forall p, \forall a \quad [13]$$

$$x^{G^*C}_{tpa} + x^{G^*G^*}_{tpa} = \pi^{G^*C}_{y,pa} y^{G^*}_{tpa} \qquad \forall t \neq T, \forall p, \forall a \quad [14]$$

$$\sum_{p\in\mathcal{P}}\sum_{a\in\mathcal{A}}\sum_{s\in\mathcal{S}} \left( \sum_{s' \in \{H,D\}} \delta^r_{0,psa} x'^{ss'}_{tpa} + \sum_{s' \notin \{H,D\}} \frac{\delta^r_s}{2} x'^{ss'}_{tpa} + \delta^r_s y^s_{tpa} \right) \leq \xi_r \qquad \forall t, \forall r \quad [15]$$

Unless stated otherwise, each parameter bound by a "$\forall$" (e.g., "$\forall t$") is assumed to range over all values of its associated set (e.g., "$\forall t$"should be read as "$\forall t \in \mathcal{T}$", whereas "$\forall p$" abbreviates "$\forall p \in \mathcal{P}$").

The model minimizes the total YLL [1] over a 1-year planning horizon (52 weeks).

In the middle of each week *t*, a new exogenous inflow of patients in need of elective care ($\phi^n_{tp}$) is observed, which adds to the cohort of waiting patients at the end of the previous week ($w_{tp}$). Note that at $t = 0$, we have a stock of patients waiting for elective care ($w_{0p}$) that have not yet been admitted to hospital. Some of these elective patients are admitted to hospital during week *t* ($z_{tpn}$); patients in need of elective care not admitted to the hospital remain in the waiting list [2]. Patients waiting for elective care are at risk of needing emergency care while waiting with probability $\pi^e_{w,p}$. These patients are immediately admitted into hospital, together with the new inflow of patients in need of emergency care ($\phi^e_{tp}$). In case of capacity shortages, admission to hospital might be denied to patients in need of

emergency care. In the model, we assume these patients ($z'^{D}_{tpe}$) die [3]. This assumption is relaxed in our sensitivity analysis in the main text.

At the moment of admission to hospital, patients may require a G&A (G) or a CC (C) bed based on the parameter $\pi^{s}_{z,tpa}$. The variables $z'^{s}_{tpa}$ define the severity state $s \in \mathcal{S}$ that patients are admitted to. Patients needing CC are assigned a C bed if available ($z'^{C}_{tpa}$); in case of capacity shortages, CC admission might be denied to a patient in need; this patient is admitted to a specific G&A state ($G^{*}$) where s/he evolves according to a new set of transition probabilities [4]. If capacity becomes available, patients in $G^{*}$ that have neither died nor been discharged are assigned a C bed. Patients needing G&A care are assigned a G bed [5].

Once in hospital, patients can transition between severity states, and they can also recover (H) or die (D). At week 0, there is already a stock of patients in hospital care ($y_0$) that corresponds to patients that were admitted to hospital prior to week 0 and that have not been discharged from hospital by then. The number of patients in a given severity state $s \in \mathcal{S}$ at the end of week $t$ ($y^{s}_{t+1,pa}$) is equal to the sum of the patients who remained in state $s$ during that week and the transitions from other states $s' \in \mathcal{S}$ to $s$ during week $t$ [6]. In particular, equations [7]-[10] and the decision variables $x'^{s's}_{tpa}$ map the transitions across severity states of newly admitted patients in their first 3.5 days of hospitalization; symmetrically, the set of equations [11]-[14] and the decision variables $x^{s's}_{tpa}$ map transitions across severity states in the following weeks.

The transition of a patient from state $s'$ to state $s$ is defined by the matrix $\pi^{s's}_{0,pa}$ for the first 3.5 days [7] and by the matrix $\pi^{s's}_{y,pa}$ [11] in the following weeks. Admission to CC is an exception to this: [8], [9], [12] and [13] enforce that in case of capacity shortages, CC might be denied to a patient in need; this patient transitions to a specific G&A state ($G^{*}$) where s/he evolves according to a new set of transition probabilities. If space in CC becomes available in the following weeks, this patient might be admitted to CC [10][14].

Patients in G&A and CC are allocated 1 bed and staff resources (nurses, doctors) based on specific staff-to-bed ratios. Equation [15] ensures that the total consumption of bed and staff resources does not exceed the available capacity ($\xi_r$). To this end, patients are divided into three distinct categories: (i) patients leaving the hospital in the first 3.5 days consume an amount of resources proportional to their length-of-stay ($\delta^{r}_{0,psa}$); (ii) newly admitted patients who remain in hospital after the first 3.5 days consume resources for a half-week ($\delta^{r}_{s}/2$); (iii) in the following weeks, patients use resources for full-week periods ($\delta^{r}_{s}$).

**Supplementary Section 2: Patient Group and Cohort Identification**

Patients are grouped using their ICD-10 root group (Supplementary Table 4) and age group (0-24, 25-64, and 65+ years). For example, ICD-10 code C15 for malignant neoplasm of the oesophagus falls under the root ICD group C00–D49: Neoplasms. This medically classifies diseases in broad categories (e.g. respiratory diseases, cancer, etc.) and thus captures any heterogeneity within the needs for emergency or elective admissions for that set of diseases.

Supplementary Table 4. ICD root group identification

| ICD-10 Chapter | Disease Category |
| --- | --- |
| A00–B99 | Certain infectious and parasitic diseases |
| C00–D48 | Neoplasms |
| D50–D89 | Diseases of the blood and blood-forming organs and certain disorders involving the immune mechanism |
| E00–E90 | Endocrine, nutritional and metabolic diseases |
| F00–F99 | Mental and behavioural disorders |
| G00–G99 | Diseases of the nervous system |
| H00–H59 | Diseases of the eye and adnexa |
| H60–H95 | Diseases of the ear and mastoid process |
| I00–I99 | Diseases of the circulatory system |
| J00–J99 | Diseases of the respiratory system |
| K00–K93 | Diseases of the digestive system |
| L00–L99 | Diseases of the skin and subcutaneous tissue |
| M00–M99 | Diseases of the musculoskeletal system and connective tissue |
| N00–N99 | Diseases of the genitourinary system |
| O00–O99 | Pregnancy, childbirth and the puerperium |
| P00–P96 | Certain conditions originating in the perinatal period |
| Q00–Q99 | Congenital malformations, deformations and chromosomal abnormalities |
| R00–R99 | Symptoms, signs and abnormal clinical and laboratory findings, not elsewhere classified |
| S00–T98 | Injury, poisoning and certain other consequences of external causes |
| V01–Y98 | External causes of morbidity and mortality |
| Z00–Z99 | Factors influencing health status and contact with health services |

Patients are further grouped into cohorts depending on the type of care needed. Patients are broadly categorized as needing emergency or elective care. For those waiting for elective care, their disease might deteriorate and consequently, these patients might need emergency treatment while waiting for elective care. Some ICD groups in our dataset, however, have too few observations to run robust empirical analyses. Thus, the ICDs representing the lowest 5% of the frequency distribution of patients in need of care separately are aggregated together for both electives and emergencies (Supplementary Table 5). This leaves 15 and 14 ICD groups for non-COVID-19 emergency admissions and elective patients in need of care, respectively, plus one COVID-19 emergency ICD group. Admitted patients are also stratified by age (0-24, 25-64 and 65+ years).

Supplementary Table 5. Bundling of elective and emergency ICDs

| Elective Bundling | Emergency Bundling |
|---|---|
| A00 – B99: Infectious and parasitic diseases | D50 – D89: Disease of blood, immune mechanism disorders |
| E00 – E89: Endocrine, nutritional, metabolic diseases | H00 – H59: Disease of eye and adnexa |
| F01 – F99: Mental, behavioural, neurodevelopment disorder | H60 – H95: Diseases of ear, mastoid process |
| H60 – H95: Diseases of ear, mastoid process | P00 – P96: Conditions originating in perinatal period |
| O00 – O99: Pregnancy, childbirth, puerperium | Q00 – Q99: Congenital malformations, deformations and chromosomal abnormalities |
| P00 – P96: Conditions originating in perinatal period | Z00 – Z99: Factors influencing health status, health services |
| Q00 – Q99: Congenital malformations, deformations, chromosomal abnormalities | |

In summary, each patient in our dataset falls into one of three cohorts: (i) non-COVID-19 patients waiting for elective care that do not require emergency treatment while waiting (Cohort A); (ii) non-COVID-19 patients in need of emergency care, including those patients that require emergency care while waiting for electives care due to progressed disease severity for the same condition for which they need elective care (Cohort B); (iii) COVID-19 patients in need of emergency care (Cohort C).

**Supplementary Section 3: Forecasting**

**3.1 Forecasting Cohorts of Hospital Care Need**

In order to estimate the number of non-COVID-19 patients in need of both elective and emergency care (i.e., the expected number of new weekly occurring patients that enter the care pathway and those that are admitted to emergency care), we fit local linear trend models with trigonometric seasonality to weekly historical data on hospital admissions for emergency patients for the various groups described in Supplementary Section 2 from January 2015 to February 2020, and we forecast from March 2020 to March 2021. For elective referrals we use weekly referrals from January 2015 to March 2019, and subsequently forecast from April 2019 to March 2021. As HES data only records a patient's referral date once the patient has been admitted to hospital, to account for the full patient cohort, we use a maximum waiting time of a year. The local linear trend model has several advantages over alternative approaches. It can deal easily with weekly seasonality, it can cope well with missing observations, it has good forecasting ability, and it can be easily interpreted.

In a local linear trend model with trigonometric seasonality,[2,3] the observed number of admissions in a specific group $y_t$ in week $t$ is decomposed into an unobserved stochastic trend $\mu_t$, an unobserved seasonal component $\gamma_t$ and an unobserved measurement error $\varepsilon_t$:

$$y_t = \mu_t + \gamma_t + \varepsilon_t \qquad \varepsilon_t \sim N(0, \sigma_\varepsilon^2).$$

The stochastic trend $\mu_t$ has the form of a unit root process with a drift which is also modelled using a unit root process:

$$\mu_{t+1} = \mu_t + \nu_t + \xi_t \qquad \xi_t \sim N\left(0, \sigma_\xi^2\right)$$
$$\nu_{t+1} = \nu_t + \zeta_t \qquad \zeta_t \sim N\left(0, \sigma_\zeta^2\right)$$

The term $\mu_t$ allows the level of the trend to change, while the drift $\nu_t$ modifies the slope of the trend over time. The seasonal term has the form:

$$\gamma_t = \sum_{j=1}^{[s/2]} \gamma_{jt},$$

where $s = 52.18$ is the period and is equal to the average number of weeks in a year accounting for leap years, and

$$\gamma_{jt+1} = \gamma_{jt} cos\lambda_j + \gamma_{jt}^* sin\lambda_j + \omega_{jt}$$
$$\gamma_{jt=1}^* = -\gamma_{jt} sin\lambda_j + \gamma_{jt}^* cos\lambda_j + \omega_{jt}^*$$
$$\lambda_j = \frac{2\,\pi\,j}{s}.$$

The parameters $\gamma_{jt}$ and $\gamma_{jt=1}^*$ capture the seasonality of the series and allow for complex seasonal patterns, while $\xi_t$, $\zeta_t$, $\omega_{jt}$ and $\omega_{jt}^*$ are white noise errors which are mutually uncorrelated. Estimation is done by maximum likelihood using the KFAS package in R.[4] Admissions needs forecasts and 95% forecasting intervals (FI) are constructed.

We forecast patients in need of care split by admission method (elective or emergency), disease group, and age band. We originally aimed to also stratify these forecasts by frailty. However, due to the small number of frail patients, we were unable to run forecasts for frail patients. Therefore, we instead forecast the proportion of patients in need of care who are frail. Analogously, while our goal was to run forecasts for patients in need of both G&A and CC (for both elective and emergency care settings), the number of patients in CC is very small for some patient groups. We therfore run forecasts for volumes of patients in need of G&A, and for proportions of patients in need of care that need CC. These proportions are forecasted using a local linear trend model with trigonometric seasonality. The dependent variable used in the estimation is not the proportion of interest $p_t$ but $y_t = \ln\left(\frac{1-p_t}{p_t}\right)$. Forecasts for $p_t$ and the corresponding forecasting intervals are obtained from forecasts and forecasting intervals of $y_t$, respectively, by $p_t = \frac{1}{1+e^{y_t}}$.

These weekly proportions are then applied to the forecasted volumes of patients in need of elective care (Cohort A) and emergency care (Cohort B) for each patient group in order to obtain the number of patients in need of care split by elective vs. emergency setting, patient group (disease, age, and frailty), and G&A vs. CC entry point.

The forecasts for the number of patients in need of care obtained from the model above tends to be accurate and to capture the seasonality of the series well. As an illustration of the forecasting performance we have estimated our time

series models over the weeks ending on 5 Jan 2015 to 6 Mar 2017 and then forecasted over the weeks ending on 13 Mar 2017 to 25 Feb 2019. Supplementary Figures 3 and 4 are two representative figures showing the forecasted and the actual admissions for two groups (elective and emergency admissions for Diseases of the digestive system, K00-K93, age group 3). Given the sensitivity of the data we cannot show graphs with individual series for small groups, but the R scripts in the GitHub repository allow to reproduce all our data when access to HES data.

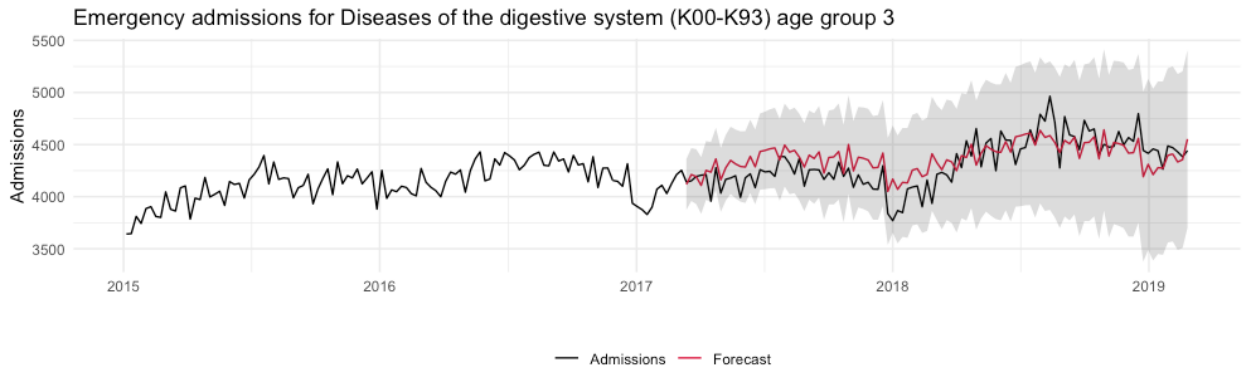


Supplementary Figure 3. Observed (black) and forecasted admission (red) for emergency in K00-K93 in age group 3. The 95% forecasting interval is in grey.

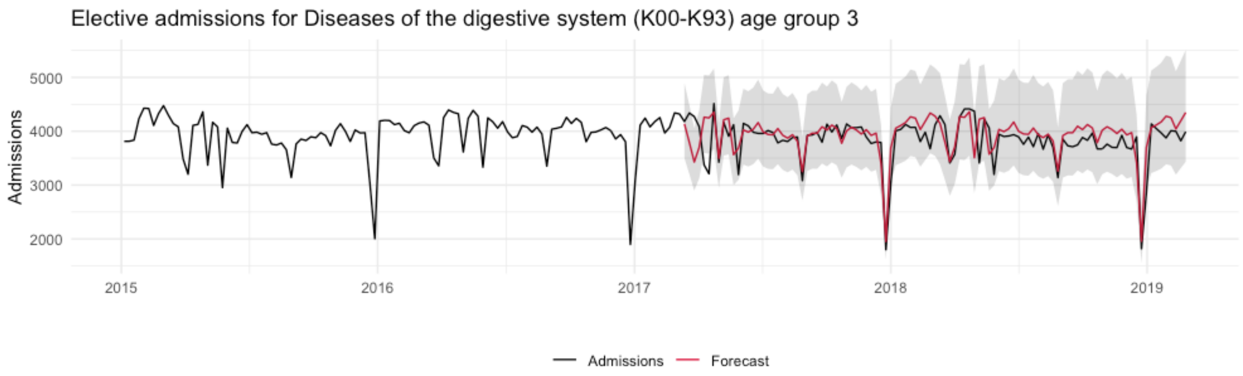


Supplementary Figure 4. Observed (black) and forecasted admission (red) for electives in K00-K93 in age group 3. The 95% forecasting interval is in grey.

### 3.2 Adjusting for Emergency Needs Reductions

Changes in care seeking behavior, changes in the prevalence of certain conditions and deaths at home have reduced the number of A&E attendances and emergency admissions during the pandemic, but our forecasts of emergency needs do not account for such changes. Therefore, we modify our forecasts for emergency admissions in the light of the changes in the patterns observed in the total hospital admission in England. In order to calculate the percentage reduction in emergency admission, we estimate a local linear trend model with trigonometric seasonality to monthly historical data (i.e., $s = 12$ in the sum of trigonometric terms) between August 2010 and February 2020. We then forecast emergency admissions and forecasting interval (FI) for the next four months and compute the percentage difference between the forecasted and the observed emergency admissions. The results are reported in Supplementary Table 6 below.

Supplementary Table 6. Forecasted versus actual emergency attendances from March to June 2020

| Month | Forecasted Emergency Attendances | Lower 95% FI | Upper 95% FI | Actual Emergency Attendances | Percentage Difference | Lower 95% FI | Upper 95% FI |
|---|---|---|---|---|---|---|---|
| March | 556,899 | 550,285 | 563,512 | 427,921 | – 30% | – 29% | – 32% |
| April | 530,080 | 523,467 | 536,694 | 326,581 | – 62% | – 60% | – 64% |
| May | 550,247 | 543,634 | 556,861 | 398,407 | – 38% | – 36% | – 40% |
| June | 534,958 | 528,344 | 541,571 | 437,535 | – 22% | – 21% | – 24% |

We found that the forecasted emergency needs reduced by around 34% during the first peak of the pandemic.

**Supplementary Section 4: Epidemiological Projections for COVID-19 Hospitalizations**

Epidemic projections are made using the integrated epidemic/economic model Daedalus,[5] in which the population consists of 4 age groups: pre-schoolers, school-age children, working-age adults, and retired. The working-age population is further divided into 63 economic sectors plus non-working adults. Each of these groups is further divided into 8 subgroups with respect to disease status: the susceptible, the exposed, the asymptomatic infectious, the infected with mild symptoms, the infected with influenza like symptoms, the hospitalized, the recovered, and the dead, whose population at time $t$ is denoted, respectively, by $S_i(t)$, $E_i(t)$, $I_i^{asym}(t)$, $I_i^{mild}(t)$, $I_i^{ILI}(t)$, $H_i(t)$, $R_i(t)$, and $D_i(t)$. Disease dynamics follow a SEIR model as follows:

$$\dot{S}_i(t) = -S_i(t)\lambda_i(t)$$
$$\dot{E}_i(t) = S_i(t)\lambda_i(t) - \sigma E_i(t)$$
$$\lambda_i(t) = \beta \sum_{j=1}^{7} M_{ij} \frac{I_j(t)}{w_j}$$
$$I_j(t) = \dot{I}_i^{asym}(t) + \dot{I}_i^{mild}(t) + \dot{I}_i^{ILI}(t)$$
$$\dot{I}_i^{asym}(t) = \sigma(1 - p_{sym})E_i(t) - \gamma_1 I_i^{asym}(t)$$
$$\dot{I}_i^{mild}(t) = \sigma p_{sym}(1 - p_{ILI})E_i(t) - \gamma_1 I_i^{mild}(t)$$
$$\dot{I}_i^{ILI}(t) = \sigma p_{sym} p_{ILI} E_i(t) - \gamma_2 I_i^{ILI}(t) - h_i I_i^{ILI}(t)$$
$$\dot{H}_i(t) = h_i I_i^{ILI}(t) - \gamma_3 H_i(t) - \mu_i H_i(t)$$
$$\dot{D}_i(t) = \mu_i H_i(t)$$
$$\dot{R}_i(t) = \gamma_1 \left(I_i^{asym}(t) + I_i^{mild}(t)\right) + \gamma_2 I_i^{ILI}(t) + \gamma_3 H(t).$$

The indices $i$ and $j$ incorporate a community of 4 age groups (0-4, 5-19, 20-64, 65+ years) and the 63 sectors of the economy, each comprising a subset of the 20-64-year-old population. The degree $x_i \in [0,1]$ to which a sector is open determines the working sector population, with $x_i = 1$ yielding the pre-lockdown scenario (fully functioning). The value of $x_i$ can be changed at discrete time intervals. All populations are subject to contacts in the community, with additional contacts made in the workplace. Opening certain sectors (schools, transport and hospitality/entertainment venues) also induces additional community contacts.

All model parameters are consistent with the real-time modelling used at Imperial College London.[6] Infections are divided into asymptomatic ("asym"), symptomatic ("sym") and influenza-like-illness ("ILI"). $P_{sym}$ denotes the proportion of infections that are symptomatic, and $p_{ILI}$ the proportion of symptomatic infections that are influenza-like. Recovery rates are denoted by the letter γ, and age-stratified hospitalization and death rates are denoted $h_i$ and $\mu_i$, respectively.

We fit four parameters to English hospital occupancy data[7] from 20th of March to 30th of June 2020, namely $t_0$ (epidemic onset), $R_0$ (basic reproduction number), $t_1$ (lockdown onset) and δ (reduction in transmission during lockdown due to NPIs). Economic closure during lockdown, $x_{min}$, is estimated from ONS,[8] alongside changes in contact rates due to working from home. For simplicity, and in order for projections to remain independent of hospitalization constraints, our projections retain the economic configuration, and we calibrate a fixed post-lockdown value of $\delta$ to yield our desired maximum value $R_{max}$ of the reproduction number $R_t$. Calibration is performed using the next-generation operator eigenvalue method,[9] for values of $R_{max} = 1.1$ and $R_{max} = 1.2$. A further value of $\delta$ is calibrated to $R_{max} = 1$ and used to impose a second lockdown on 1st December 2020 (Early LD) or 1st January 2021 (Late LD). This second lockdown is exogenous to the hospital capacity model, whilst representing a realistic mitigation strategy. We therefore have four different scenarios, comprising all combinations of $R_{max}$ and onset of second lockdown.

Supplementary Figure 5 presents the fitted initial epidemic and 4 projected scenarios.

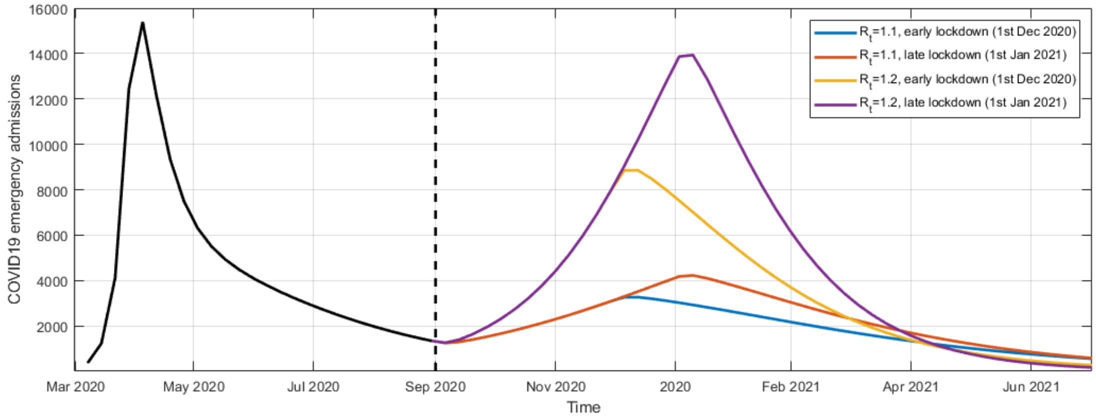

Supplementary Figure 5. Fitted initial epidemic and 4 projected scenarios.

**Supplementary Section 5: Transition Probabilities**

We estimate transition probabilities for both patients waiting to receive care and those admitted to hospital.

### 5.1. Patients in Need of Emergency Care due to Prolonged Waiting to Receive Care ($\pi^{e}_{wp}$)

It is possible that patients waiting for elective care need emergency care due to prolonged waiting times imposed by prioritization rules and/or the various scenarios (e.g. postponement of elective admissions lead to delayed access to care for those patients). The longer patients wait, the more likely they are to need emergency care. We identify these types of patients as those that had an emergency admission for the same ICD as their elective admission while they were waiting for elective care. Specifically, we calculate the waiting period as the difference in time between the date (*rttperstart_elective* in HES) the patient entered the care pathway (i.e. when they are first referred to a consultant for a new condition) and the date the patient is admitted for emergency care for the same ICD (*admidate_emergency* in HES).

For our optimization model in Supplementary Section 1, we calculate the probability ($\pi_p$) that an individual within patient group $p$ who is not admitted as an elective in a certain week may be admitted as an emergency in that week conditional on having already waited for a certain time period:

$$Pr\{WT \leq wt + 1 | WT \geq wt\}$$

where $WT$ is the length of time between the referral to treat date and the emergency admission and $wt$ is the length of time the individual has already waited pre-admission. The probability of switching in week $wt + 1$ is calculated using survival analysis methods as:

$$Pr\{WT \leq wt + 1 \mid WT \geq wt\} = \frac{1 - Pr\{WT \geq wt + 1\}}{Pr\{WT \geq wt\}} = \frac{1 - S(wt + 1)}{S(wt)},$$

where $S(t), t \geq 0,$ is the survival function. Assuming we have independent and identically distributed observations for $N$ individuals with censoring time independent of the survival time, the required probability can be estimated using $\frac{1-\hat{S}(wt+1)}{\hat{S}(wt)}$, where $\hat{S}(wt)$ is the Kaplan-Meier estimator of the survival function. The estimation is done using the package Survival in R.[10] We calculate these probabilities at weekly intervals of waiting time over a ten-week period (i.e. *wt* = 7, 14, 21 ,..,70). Then the average of these probabilities is used as input into the optimization model to give an estimate of this incidence across a range of *wt*.

Ideally, we would have estimated the transition probabilities stratified for every patient group defined by the ICDs, age group and frailty score outlined in Supplementary Section 2. However, due to small sample sizes in some of those groups, Kaplan-Meier estimates would not be precise. Therefore, we bundle some ICDs together for both emergency and elective admissions. Specifically, we bundle the ICDs representing the lowest 5% of the frequency distribution of electives as well as emergency patients in need of care. While the same criterion is applied for electives and for emergencies, the bundled groups do not match between electives and emergencies (Supplementary Table 5). Thus, in estimating the transition probabilities we pool all patients in the elective bundle. Then we further refine that transition probability by combining it with the forecasted proportions of each ICD/age group out of the total number of patients in the bundle. The latter is accomplished using a local linear trend model with trigonometric seasonality subject to suitable transformations to account for the fact that a proportion is between zero and one (Supplementary Section 3.1). In particular, the probability of transitioning from waiting for elective care to needing emergency care is applied to the cohort of patients in need of elective care (Cohort A). Then, we apply the forecasted proportions who move from the electives to the emergency bundle of patients to this newly calculated stock of patients transitioning, in order to ascertain the number of patients in each ICD group that move from needing elective (Cohort A) to emergency (Cohort B) care.

### 5.2. Patients Admitted to Hospital ($\pi^{ss'}_{y,ap}$)

Upon admission to hospital, patients in need of elective and emergency care can move between different states in their immediate care pathway. A patient can be admitted to G&A, then transition to being discharged (i.e. recovery), CC or die. Similarly, a patient can be admitted to CC (either directly or through G&A), then transition into recovery,

(return) to G&A or die. For each G&A and CC starting state, the end states are mutually exclusive and jointly exhaustive outcomes.

Most patients admitted to G&A or CC beds stay for less than a week. Since our time unit is a week, estimating the transition probabilities at the end of the week would lead to an over-estimate of the patients' expected time in hospital. Therefore, we split the first week in half and estimate the transition probabilities for patients in the first half of the week (i.e., at 3.5 days from admission). For patients that remain in G&A longer than 3.5 days we estimate the probability of dying, being discharged from hospital, being transferred to CC or continue staying in a G&A bed by the end of day 10.5 of their admission. In the optimization model, the transition probabilities at 3.5 days are used as transition probabilities at the end of the first week from admissions. The transition probabilities at 10.5 days are then used for all subsequent weeks. For the stock of patients already in hospital at the start of the pandemic, the transition probabilities at 10.5 days apply.

The transition probabilities at 3.5 days are estimated using a multinomial logit. We estimate the transition probabilities at 10.5 days using a multinomial logit for individuals who stay longer than 3.5 days.

We make the following assumptions in this analysis.

First, patients in need of emergency care, both non-COVID-19 (Cohort B) and COVID-19 (Cohort C), are admitted without waiting. Therefore, their in-hospital transition probabilities are not conditioned on waiting time. For patients admitted for elective care, the transition probabilities are estimated conditional on waiting times.

Second, we do not include patients that need emergency care while waiting for elective procedures in the calculations of elective (Cohort A) transition probabilities. Thus, it is possible that we underestimate some of the probabilities of transitioning into more severe states (e.g. CC or death).

Third, in the absence of data during the COVID-19 epidemic, we assume that the in-hospital transition probabilities estimated for non-COVID-19 patients pre-pandemic remained unchanged after the start of the pandemic.

Fourth, given the above assumption may be reasonable when hospital capacity is far from being exhausted but too strong under full capacity constraints (i.e., hospitals operating at capacity will see patients who would normally be admitted to CC remaining in G&A beds), we make the following simplifying assumptions for non-COVID-19 patients denied CC (these are the patients in group $G^*$):

(i) The probability of an individual $i$ dying at time $t$ if denied CC when in need is at least as large as the probability of dying if timely admitted to CC such that:

$$Pr_t^i\{dying|denied\ CC\} = \frac{1}{2}\left(Pr_t^i\{dying|CC\} + 1\right)$$

(ii) The probability that a patient who has been denied CC, is discharged alive is half of the smaller between the probability of not dying if denied CC and the probability that a patient who is in CC is first discharged to G&A and then discharged alive:

$$Pr_t^i\{discharged\ alive|denied\ CC\} = \frac{1}{2}min\left\{1 - Pr_t^i\{dying|denied\ CC\}, Pr_t^i\{discharged\ alive|G\&A\}\ Pr_t^i\{G\&A|in\ CC\}\right\}$$

(iii) The probability of an individual $i$ remaining in G&A at time step $t$ is 1 minus both of the above probabilities:

$$Pr_t^i\{G\&A|denied\ CC\} = 1 - Pr_t^i\{dying|denied\ CC\} - Pr_t^i\{discharged\ alive|denied\ CC\}.$$

Patients initially denied CC are in need of CC in subsequent weeks and can therefore be admitted to CC at all subsequent weekly time steps if capacity becomes available. If capacity does not become available, these patients will stay in G&A and the transition probabilities conditional on being denied CC apply to them.

Lastly, for the case of COVID-19 patients, we calculate the above transition probabilities as previously described directly from the available data.

**Supplementary Section 6: Costs and Years of Life Lost**

**6.1 Estimating Unit Costs**

To calculate the cost of care provided in hospital, we link HES data with reference costs via their HRG. Patients are first matched via hospital and HRG using 2018-19 organizational reference cost data. If they could not be matched, they are then linked via just the HRG using the 2018-19 national reference cost schedule. We attempt to match any remaining unmatched patients on either hospital and HRG using organizational or national reference cost data from 2017-18, then 2016-17, and then 2015-16. Due to data limitations, the majority of HES data could not be costed in years 2015-17. We therefore calculate average costs per patient group using admissions from 2017-19, where only fewer than 1% of admissions are not costed. There are no significant differences between average costs calculated using 2017-19 data and 2015-19 data. While it is possible that our cost estimates may be biased due to these missing matched patients, the small percentage of unmatched patients suggests that this bias is likely negligible.

Since HRGs do not yet exist for COVID-19 patients, we estimate their hospitalization costs by building our own HRGs using the HRG4+ 2020/21 Local Payment Grouper publicly available from NHS Digital.[11] The grouper is a computer program that assigns an HRG by considering various patient-level information and is the same software used by the NHS to generate HRGs in the HES data. We therefore take the following individual-level information from administrative discharge records of COVID-19 patients from ICHNT and feed them into the grouper to create individual HRGs for each patient: managing hospital, area of admission (clinical vs surgical), age, sex, method of admission (emergency vs elective), discharge destination, length of stay (days), number of consultant assessment episodes, list of final diagnoses (ICD-10) and procedures (OPCS-4), among others. An average unit cost as well as distributions of unit costs per patient group are then calculated for each cohort.

**6.2 Years of Life Lost**

To calculate the unit YLL for each age group, we take the unweighted average of the age specific life expectancy across all ages in that group. The unit YLL per death for each age group is subsequently multiplied by the number total number of deaths of the group (irrespective of the age distribution if the patients are within the group) estimated by the optimization model to provide the total YLL.

This is summarized using the equation below:

$$YLL_i = (Life\ Expectancy - Age\ Group\ midpoint_i) \times Total\ number\ of\ deaths_i$$

with $i = \{< 25, 25 - 64, 64\}$ denoting the age group.

As a sensitivity analysis, we also calculate the unit YLL in the following way. Using the life expectancy (LE) at birth for the UK in 2020 (81.15 years),[12] we derive the unit YLL per death for each age group by taking the difference between LE and the midpoint of the age group (i.e., at 12.5, 44.5 and 73 years). For example, for the age group 65+, the YLL per death is 8 (i.e., 81.15 –73, where 73 is the midpoint of the age group). Supplementary Table 7 below summarizes the unit YLL per death across the age groups considered.

Supplementary Table 7. Unit YLL per death across the three age categories

| Age Group *i* | Unit YLL per death using LE at birth | Unit YLL per death using age specific LE |
|---|---|---|
| <25 | 68.85 | 69.9 |
| 25-64 | 36.65 | 38.5 |
| 65+ | 8.15 | 8.9 |

**Supplementary Section 7: Standard Policies**

We use the following *Standard Policies* to mimic the prioritization of non-frail patients to CC and the postponement of scheduled elective procedures that has been implemented by the English government.

On the 20th of March 2020, England's National Institute for Health and Care Excellence (NICE) published a critical care prioritization guideline for adults during the COVID-19 pandemic.[13] The guidelines suggest how to prioritize admission of adult patients to critical care. In general, NICE suggested to assess each patient using a frailty assessment according to age. Those over 65 years of age without long-term disabilities, learning disabilities, or autism are to be assessed using the Clinical Frailty Scale (CFS) score. Physicians are suggested to use an individualized assessment of frailty and not the CFS score for patients under 65-years-old with long-term disabilities, learning disabilities, or autism. Those who are deemed to be less frail (e.g., CFS score < 5) and would like CC treatment would be referred to CC if their condition worsened. Those who are identified as frailer (e.g., CFS score of 5+) are further assessed whether CC was appropriate (measures undefined by NICE; presumably left to the physicians' discretion). If so, then these patients could still be admitted to CC if their condition deteriorated. If not, then these patients would receive end-of-life care.

To mirror this policy change, we include a prioritization rule to CC whereby in weeks where CC capacity is full, patients are prioritized based on their frailty score. That is, patients who are not frail are prioritized over frail patients.

Furthermore, in a letter to NHS staff on the 17th of March 2020, NHS England's Chief Executive and the NHS Chief Operating Officer informed hospitals to cancel all non-urgent elective operations from April 15th at the latest. This was implemented for hospitals to free up the maximum possible capacity in anticipation of upcoming surges in demand due to COVID-19 patients. On the 23rd of April, 2020, it was announced that hospitals should re-start other services.[14] Therefore, we leverage these decisions to model four *Standard Policies*.

In *Standard Policy 1*, we mimic the implementation of the policy described above that occurred in England between 17th of March and 23rd of April (weeks 3-8) and that consists of prioritization of patients to critical care based on frailty and postponement of non-urgent elective operations. *Standard Policy 1* assumes that the policy was only enacted during the actual time-period (weeks 3-8) and the postponement of 100% of elective procedures. *Standard Policy 2* considers potential policy implementation during future pandemic peaks by switching the policy on and off depending on the number of predicted COVID-19 cases in the population. We switch the policy on (postponement of 100% of electives) when the number of predicted COVID-19 cases surpass 4,118 (the observed number of cases on 17th of March). The policy is switched off when the number of predicted COVID-19 hospitalizations begins to decline and falls below 7,494 (the observed number of cases on 23rd of April). If the number of cases never reaches the peak 7,494 after the policy is switched on, then it is switched off when the number of hospitalizations begins to decrease. Therefore, for $R_t = 1.1$, the policy switches on between weeks 3-8 and 44-50 for Late Lockdown and between weeks 3-8 only for Early Lockdown. Likewise, for $R_t = 1.2$, the policy switches on between weeks 3-8 and 35-54 for Late Lockdown and between weeks 3-8 and 35-44 for Early Lockdown. *Standard Policies 3 and 4* follow the same rules as *Standard Policies 1 and 2* except with the postponement of 75% of electives.

For all policies, we seperately consider each of the different epidemiological scenarios, namely: Baseline (Early and Late Lockdown), Best-Case (Early and Late Lockdown) and Worst-Case scenario (Early and Late Lockdown).

**7.1 Simulation Model**

We develop a simulation model over a 52-week planning-horizon to replicate the *Standard Policies* and compare their outcomes against those of the *Optimized Schedules*.

The simulation model admits patients to hospital according to a rule-based system, by which patients are admitted to hospital according to their order of priority as determined pre-pandemic; in addition to this, they account for the postponement of a fraction of elective admissions over given weeks of the planning horizon. For each scenario, we implement a postponement of "x%" elective admissions during the weeks in which the *Standard Policy* is activated. Patients for which their elective procedures are postponed remain in the queue awaiting admission at the earliest possible time according to a FIFO rule. We consider two values for "x%", namely 100% (in *Standard Policies 1 and 2*) and 75% (in *Standard Policies 3 and 4*). In addition, during the weeks in which the *Standard Policy* is on, CC is prioritized for non-frail patients (emergency and elective) of each patient group.

**7.1 Model Inputs**
At the beginning of the time horizon ($t = 0$) we have as inputs the total available resources and an initial stock of patients comprising of patients hospitalized at $t = 0$ in CC and G&A, and elective patients awaiting admission. We aggregate patients by disease type and severity state, allowing for enough differentiation to closely reflect the individual patient characteristics. For each subgroup, we also have information detailing their resource requirements, transition probabilities, and frailty proportions. The transition probabilities represent the evolution of a patient's condition (reflected by their severity state) once admitted to hospital, while the frailty proportions for each group are used for prioritizing access to CC for non-frail patients during the time periods that the resources are rationed. For $t > 0$, based on the scenario we are investigating (e.g., reproduction number, Late vs. Early Lockdown), we observe new exogenous inflows of patients. Moreover, for each *Standard Policy* that is implemented, we have as input the time period in which the *Policy* is activated.

**7.2 Model Assumptions**
We impose the following assumptions on the simulation model:

(i) If an incoming emergency patient is denied access to hospital due to shortage of beds in G&A at a particular time period, we assume that the patient dies if no more emergcncy capacity is created (Upper Bound case) or the patient is seen in extra emergency capacity created by the government (Lower Bound case).
(ii) The non-frail prioritization rule during the weeks in which the policy is activated applies only to new incoming patients requiring CC. That is, if a patient is in CC the week before the *Standard Policy* is turned on (week 2) and this patient again requires CC in the week that the *Standard Policy* is turned on (week 3), this patient will not be removed from CC even though s/he may be frail.
(iii) During the weeks in which the *Standard Policy* is on, non-frail patients are prioritized for access to CC. However, if beds are available once all the non-frail patients have been admitted to CC, the remaining beds are allocated to frail patients.
(iv) With regards to access to resources, a patient already in hospital has higher priority over incoming patients. That is, no patient already admitted in hospital is removed from hospital to make space for an incoming patient.

**7.3 Model Implementation**
At the beginning of each week/time period $t$ (where $0 \leq t \leq T$), all resources are available to the model. First, patients currently in hospital from the previous week transition from their state in week $t - 1$ to their current state in week $t$. The transition occurs according to a Markov Chain which uses the transition probabilities of the subgroup that the patient belongs to. If a patient recovers (i.e., transitions to "H" state), s/he is removed from the system. If a patient dies (i.e., transitions to "D" state), the corresponding YLL is updated and the patient is removed from the system. For any other transition, patients are moved or allowed to stay on in the ward where they require care, and the resources required in the current week are updated accordingly. Once all the patients have transitioned, we check if the resource requirement in CC for the current week exceeds the resource availability in CC. If this is the case, we choose uniformly among patients needing CC and move them to G&A (G*) until the resources in CC are no longer over-utilized. By design, we always have enough resources in G&A to accommodate patients already in hospital from previous weeks.

We next make admissions decisions regarding the patients in need of emergency care arising from the inflows (of standard emergencies as well as patients in need of emergency care as a result of waiting for elective care) in the

current week. We first handle the emergency patients requiring CC, followed by those requiring G&A. Our handling of these patients depends on whether the *Standard Policy* is on/off in the current week.

(i) *When the Standard Policy is on:* The non-frail patients from each patient subgroup are prioritized for access to CC. If not enough resources in CC are available to accommodate all non-frail patients requiring CC, we employ uniform sampling to choose non-frail patients across subgroups that are admitted to CC for the current week. The remaining patients are allocated G&A, where they evolve according to a new set of transition probabilities (G*). However, if space remains available in CC once all the non-frail patients have been accommodated in CC, the remaining resources are uniformly distributed amongst the frail patients belonging to the different subgroups. The remaining frail patients (if any) are moved to G&A (G*). Finally, we admit patients that require G&A. If resources in G&A are insufficient, we once again employ uniform sampling across subgroups to admit patients to G&A. Any emergency patients denied admission to G&A are assumed to either die if no more emergency capacity is created (Upper Bound case) or to be seen in extra emergency capacity created by the government (Lower Bound case).

(ii) *When the Standard Policy is off:* In these weeks, the mechanism for admissions remains the same as in the weeks when the *Standard Policy* is on, except that the patients are not prioritized by frailty for access to CC.

Finally, we make decisions regarding the elective admissions for the current week. The patients are first added to the waiting queue (which may be empty) corresponding to their subgroups. Subsequently, the patients are admitted from the queue according to a FIFO rule. That is, we first admit patients who entered the waiting queue at an earlier point in time before admitting patients who entered the queue at a later time.

(i) *When the Standard Policy is on:* In these weeks, we postpone x% of electives. That is, we can only admit up to (100 - x)% of the electives given that there is sufficient space available. Moreover, in these weeks, CC is prioritized for non-frail patients belonging to each subgroup. The number of patients admitted in the current week are thus driven by the *Standard Policy* and the availability of resources. The number of patients admitted from each subgroup is proportional to the fraction of the total waiting patients belonging to that particular subgroup.

(ii) *When the Standard Policy is off:* In these weeks, we admit as many elective patients as can be accommodated in CC and G&A subject to availability of resources. As in (i), the number of patients admitted from each subgroup is proportional to the fraction of the total waiting patients belonging to that particular subgroup.

**7..4 Model Outputs**

At each time period, for each subgroup of patients we track the following:

(i) number of elective and emergency admissions made, and the associated costs;
(ii) contribution to YLL as a result of patients dying;
(iii) emergency patients denied admission to hospital;
(iv) the beds utilized in CC and G&A (differentiated by those that require G&A and those that require CC but were moved to G&A because of insufficient beds in CC).

**Supplementary Section 8: Simulation & Scenarios for Optimized Schedule and Standard Policies**

Supplementary Table 8. Constraints and assumptions for each scenario and simulation

| Simulation | Scenario Name | X% Postponement of Electives, Policy Description | Reproduction Number ($R_t$)/Lockdown month | Capacity | Emergency Care-Seeking Behavior |
|---|---|---|---|---|---|
| Optimized Schedule | Baseline Early Lockdown | N/A | 1.1/Dec | Normal | Normal |
| | Baseline Late Lockdown | N/A | 1.1/Feb | Normal | Normal |
| | Best-Case Early Lockdown | N/A | 1.1/Dec | Expanded | Reduced |
| | Best-Case Late Lockdown | N/A | 1.1/Feb | Expanded | Reduced |
| | Worst-Case Early Lockdown | N/A | 1.2/Dec | Normal | Normal |
| | Worst-Case Late Lockdown | N/A | 1.2/Feb | Normal | Normal |
| Standard Policy 1 | Baseline Early Lockdown | 100%, on over weeks 3-8 | 1.1/Dec | Normal | Normal |
| | Baseline Late Lockdown | 100%, on over weeks 3-8 | 1.1/Feb | Normal | Normal |
| | Best-Case Early Lockdown | 100%, on over weeks 3-8 | 1.1/Dec | Expanded | Reduced |
| | Best-Case Late Lockdown | 100%, on over weeks 3-8 | 1.1/Feb | Expanded | Reduced |
| | Worst-Case Early Lockdown | 100%, on over weeks 3-8 | 1.2/Dec | Normal | Normal |
| | Worst-Case Late Lockdown | 100%, on over weeks 3-8 | 1.2/Feb | Normal | Normal |
| Standard Policy 2 | Baseline Early Lockdown | 100%, on/off with thresholds | 1.1/Dec | Normal | Normal |
| | Baseline Late Lockdown | 100%, on/off with thresholds | 1.1/Feb | Normal | Normal |
| | Best-Case Early Lockdown | 100%, on/off with thresholds | 1.1/Dec | Expanded | Reduced |
| | Best-Case Late Lockdown | 100%, on/off with thresholds | 1.1/Feb | Expanded | Reduced |
| | Worst-Case Early Lockdown | 100%, on/off with thresholds | 1.2/Dec | Normal | Normal |
| | Worst-Case Late Lockdown | 100%, on/off with thresholds | 1.2/Feb | Normal | Normal |
| Standard Policy 3 | Baseline Early Lockdown | 75%, on over weeks 3-8 | 1.1/Dec | Normal | Normal |
| | Baseline Late Lockdown | 75%, on over weeks 3-8 | 1.1/Feb | Normal | Normal |
| | Best-Case Early Lockdown | 75%, on over weeks 3-8 | 1.1/Dec | Expanded | Reduced |
| | Best-Case Late Lockdown | 75%, on over weeks 3-8 | 1.1/Feb | Expanded | Reduced |
| | Worst-Case Early Lockdown | 75%, on over weeks 3-8 | 1.2/Dec | Normal | Normal |
| | Worst-Case Late Lockdown | 75%, on over weeks 3-8 | 1.2/Feb | Normal | Normal |
| Standard Policy 4 | Baseline Early Lockdown | 75%, on/off with thresholds | 1.1/Dec | Normal | Normal |
| | Baseline Late Lockdown | 75%, on/off with thresholds | 1.1/Feb | Normal | Normal |
| | Best-Case Early Lockdown | 75%, on/off with thresholds | 1.1/Dec | Expanded | Reduced |
| | Best-Case Late Lockdown | 75%, on/off with thresholds | 1.1/Feb | Expanded | Reduced |
| | Worst-Case Early Lockdown | 75%, on/off with thresholds | 1.2/Dec | Normal | Normal |
| | Worst-Case Late Lockdown | 75%, on/off with thresholds | 1.2/Feb | Normal | Normal |

**Supplementary Section 9: Results - Figures and Tables**

Supplementary Table 9. Health economic metrics for Optimized Schedule and Standard Policy scenarios

| Simulation | Scenario Name | Total Cost (£) Lower Bound | Total Cost (£) Upper Bound | Unit Cost (£) Lower Bound | Unit Cost (£) Upper Bound | Total YLL Lower Bound | Total YLL Upper Bound | Unit YLL Lower Bound | Unit YLL Upper Bound |
|---|---|---|---|---|---|---|---|---|---|
| Optimized Schedule | Baseline Early Lockdown | 23,172,645,322 | 23,180,277,221 | 2,593 | 2,593 | 5,113,010 | 5,128,676 | 0.57 | 0.57 |
| | Baseline Late Lockdown | 23,260,492,311 | 23,268,124,210 | 2,594 | 2,594 | 5,349,583 | 5,365,249 | 0.60 | 0.60 |
| | Best-Case Early Lockdown | 20,458,090,671 | 20,458,090,671 | 2,452 | 2,452 | 4,313,168 | 4,313,168 | 0.52 | 0.52 |
| | Best-Case Late Lockdown | 20,525,014,239 | 20,525,014,239 | 2,454 | 2,454 | 4,548,245 | 4,548,245 | 0.54 | 0.54 |
| | Worst-Case Early Lockdown | 23,196,719,457 | 23,204,351,356 | 2,595 | 2,596 | 6,062,658 | 6,078,324 | 0.68 | 0.68 |
| | Worst-Case Late Lockdown | 22,875,896,932 | 23,151,034,351 | 2,594 | 2,625 | 7,265,807 | 7,802,635 | 0.82 | 0.88 |
| Standard Policy 1 | Baseline Early Lockdown | 23,586,092,197 | 23,610,654,035 | 2,629 | 2,632 | 5,432,412 | 5,687,338 | 0.61 | 0.63 |
| | Baseline Late Lockdown | 23,376,881,996 | 23,400,849,801 | 2,641 | 2,643 | 5,669,329 | 5,918,189 | 0.64 | 0.67 |
| | Best-Case Early Lockdown | 20,505,687,124 | 20,505,687,124 | 2,456 | 2,456 | 4,374,622 | 4,374,622 | 0.52 | 0.52 |
| | Best-Case Late Lockdown | 20,572,656,470 | 20,572,656,470 | 2,458 | 2,458 | 4,598,994 | 4,598,994 | 0.55 | 0.55 |
| | Worst-Case Early Lockdown | 22,921,627,767 | 22,974,001,499 | 2,667 | 2,673 | 6,580,820 | 7,129,868 | 0.77 | 0.83 |
| | Worst-Case Late Lockdown | 22,542,522,425 | 23,096,863,762 | 2,679 | 2,744 | 7,863,702 | 13,694,243 | 0.93 | 1.63 |
| Standard Policy 2 | Baseline Early Lockdown | 23,586,092,197 | 23,610,654,035 | 2,629 | 2,632 | 5,432,412 | 5,687,338 | 0.61 | 0.63 |
| | Baseline Late Lockdown | 23,142,500,909 | 23,169,518,571 | 2,649 | 2,652 | 5,644,358 | 5,928,651 | 0.65 | 0.68 |
| | Best-Case Early Lockdown | 20,505,687,124 | 20,505,687,124 | 2,456 | 2,456 | 4,374,622 | 4,374,622 | 0.52 | 0.52 |
| | Best-Case Late Lockdown | 20,578,064,541 | 20,578,064,541 | 2,459 | 2,459 | 4,608,359 | 4,608,359 | 0.55 | 0.55 |
| | Worst-Case Early Lockdown | 22,642,100,874 | 22,700,123,931 | 2,678 | 2,685 | 6,533,706 | 7,141,595 | 0.77 | 0.84 |
| | Worst-Case Late Lockdown | 22,284,356,892 | 22,856,483,668 | 2,689 | 2,758 | 7,822,713 | 13,846,478 | 0.94 | 1.67 |
| Standard Policy 3 | Baseline Early Lockdown | 23,584,191,269 | 23,613,900,867 | 2,629 | 2,632 | 5,422,959 | 5,731,883 | 0.60 | 0.64 |
| | Baseline Late Lockdown | 23,440,849,623 | 23,467,680,979 | 2,638 | 2,641 | 5,670,197 | 5,951,270 | 0.64 | 0.67 |
| | Best-Case Early Lockdown | 20,495,121,622 | 20,495,121,622 | 2,455 | 2,455 | 4,388,301 | 4,388,301 | 0.53 | 0.53 |
| | Best-Case Late Lockdown | 20,561,970,208 | 20,561,970,208 | 2,457 | 2,457 | 4,638,655 | 4,638,655 | 0.55 | 0.55 |
| | Worst-Case Early Lockdown | 23,000,918,683 | 23,065,495,500 | 2,663 | 2,670 | 6,552,316 | 7,228,642 | 0.76 | 0.84 |
| | Worst-Case Late Lockdown | 22,640,593,809 | 23,196,178,229 | 2,674 | 2,739 | 7,857,464 | 13,686,998 | 0.93 | 1.62 |
| Standard Policy 4 | Baseline Early Lockdown | 23,584,191,269 | 23,613,900,867 | 2,629 | 2,632 | 5,422,959 | 5,731,883 | 0.60 | 0.64 |
| | Baseline Late Lockdown | 23,289,336,491 | 23,323,140,112 | 2,643 | 2,647 | 5,662,916 | 6,015,280 | 0.64 | 0.68 |
| | Best-Case Early Lockdown | 20,495,121,622 | 20,495,121,622 | 2,455 | 2,455 | 4,388,301 | 4,388,301 | 0.53 | 0.53 |
| | Best-Case Late Lockdown | 20,565,134,573 | 20,565,134,573 | 2,457 | 2,457 | 4,613,766 | 4,613,766 | 0.55 | 0.55 |
| | Worst-Case Early Lockdown | 22,747,930,816 | 22,808,212,960 | 2,673 | 2,681 | 6,584,569 | 7,211,577 | 0.77 | 0.85 |
| | Worst-Case Late Lockdown | 22,443,227,051 | 23,011,823,995 | 2,682 | 2,750 | 7,812,586 | 13,800,274 | 0.93 | 1.65 |

Supplementary Table 10. ICER calculations comparing Optimized Schedule and Standard Policy scenarios

| Simulation | Scenario Name | Incremental YLG Lower Bound | Incremental YLG Upper Bound | Incremental Costs Lower Bound | Incremental Costs Upper Bound | ICER (£ per YLG) Lower Bound | ICER (£ per YLG) Upper Bound |
|---|---|---|---|---|---|---|---|
| Optimized Schedule vs. Standard Policy 1 | Baseline Early Lockdown | 319,402 | 558,662 | -£430,376,814 | -£413,446,875 | Optimization Dominates | Optimization Dominates |
| | Baseline Late Lockdown | 319,747 | 552,940 | -£132,725,591 | -£116,389,685 | Optimization Dominates | Optimization Dominates |
| | Best-Case Early Lockdown | 61,454 | 61,454 | -£47,596,452 | -£47,596,452 | Optimization Dominates | Optimization Dominates |
| | Best-Case Late Lockdown | 50,750 | 50,750 | -£47,642,231 | -£47,642,231 | Optimization Dominates | Optimization Dominates |
| | Worst-Case Early Lockdown | 518,162 | 1,051,544 | £230,349,858 | £275,091,690 | £445 | £262 |
| | Worst-Case Late Lockdown | 597,895 | 5,891,608 | £54,170,589 | £333,374,507 | £91 | £57 |
| Optimized Schedule vs. Standard Policy 2 | Baseline Early Lockdown | 319,402 | 558,662 | -£430,376,814 | -£413,446,875 | Optimization Dominates | Optimization Dominates |
| | Baseline Late Lockdown | 294,776 | 563,403 | £98,605,639 | £117,991,402 | £335 | £209 |
| | Best-Case Early Lockdown | 61,454 | 61,454 | -£47,596,452 | -£47,596,452 | Optimization Dominates | Optimization Dominates |
| | Best-Case Late Lockdown | 60,114 | 60,114 | -£53,050,302 | -£53,050,302 | Optimization Dominates | Optimization Dominates |
| | Worst-Case Early Lockdown | 471,048 | 1,063,271 | £504,227,425 | £554,618,584 | £1,070 | £522 |
| | Worst-Case Late Lockdown | 556,905 | 6,043,843 | £294,550,683 | £591,540,040 | £529 | £98 |
| Optimized Schedule vs. Standard Policy 3 | Baseline Early Lockdown | 309,949 | 603,207 | -£433,623,646 | -£411,545,947 | Optimization Dominates | Optimization Dominates |
| | Baseline Late Lockdown | 320,614 | 586,021 | -£199,556,768 | -£180,357,312 | Optimization Dominates | Optimization Dominates |
| | Best-Case Early Lockdown | 75,133 | 75,133 | -£37,030,951 | -£37,030,951 | Optimization Dominates | Optimization Dominates |
| | Best-Case Late Lockdown | 90,410 | 90,410 | -£36,955,969 | -£36,955,969 | Optimization Dominates | Optimization Dominates |
| | Worst-Case Early Lockdown | 489,658 | 1,150,318 | £138,855,856 | £195,800,775 | £284 | £170 |
| | Worst-Case Late Lockdown | 591,657 | 5,884,363 | -£45,143,878 | £235,303,123 | Optimization Dominates | Optimization Dominates |
| Optimized Schedule vs. Standard Policy 4 | Baseline Early Lockdown | 309,949 | 603,207 | -£433,623,646 | -£411,545,947 | Optimization Dominates | Optimization Dominates |
| | Baseline Late Lockdown | 313,334 | 650,031 | -£55,015,902 | -£28,844,180 | Optimization Dominates | Optimization Dominates |
| | Best-Case Early Lockdown | 75,133 | 75,133 | -£37,030,951 | -£37,030,951 | Optimization Dominates | Optimization Dominates |
| | Best-Case Late Lockdown | 65,521 | 65,521 | -£40,120,334 | -£40,120,334 | Optimization Dominates | Optimization Dominates |
| | Worst-Case Early Lockdown | 521,911 | 1,133,252 | £396,138,396 | £448,788,642 | £759 | £396 |
| | Worst-Case Late Lockdown | 546,779 | 5,997,639 | £139,210,355 | £432,669,881 | £255 | £72 |

Supplementary Table 11. Patient flow metrics for Optimized Schedule and Standard Policy scenarios

| Simulation | Scenario Name | Total Elective Admissions | COVID Emergency Admissions | Non-COVID Emergency Admissions | Total Emergency Admissions | Total Admissions | Waiting Patients | Total Admission Denials |
|---|---|---|---|---|---|---|---|---|
| Optimized Schedule | Baseline Early Lockdown | 2,204,773 | 179,349 | 6,553,861 | 6,733,210 | 8,937,983 | 1,531,063 | 2,240 |
| | Baseline Late Lockdown | 2,228,839 | 199,681 | 6,540,015 | 6,739,697 | 8,968,536 | 1,520,843 | 2,240 |
| | Best-Case Early Lockdown | 3,868,494 | 181,955 | 4,294,432 | 4,476,386 | 8,344,881 | 0 | 0 |
| | Best-Case Late Lockdown | 3,868,977 | 202,287 | 4,293,949 | 4,496,235 | 8,365,213 | 0 | 0 |
| | Worst-Case Early Lockdown | 2,156,243 | 249,400 | 6,533,075 | 6,782,474 | 8,938,718 | 1,600,380 | 2,240 |
| | Worst-Case Late Lockdown | 2,021,097 | 311,406 | 6,487,094 | 6,798,500 | 8,819,596 | 1,744,954 | 74,099 |
| Standard Policy 1 | Baseline Early Lockdown | 2,153,733 | 180,997 | 6,635,396 | 6,816,393 | 8,970,126 | 1,487,913 | 8,630 |
| | Baseline Late Lockdown | 2,013,659 | 201,339 | 6,638,145 | 6,839,484 | 8,853,143 | 1,625,370 | 8,419 |
| | Best-Case Early Lockdown | 3,849,717 | 182,036 | 4,317,975 | 4,500,011 | 8,349,728 | 0 | 0 |
| | Best-Case Late Lockdown | 3,849,739 | 202,363 | 4,317,954 | 4,520,317 | 8,370,056 | 0 | 0 |
| | Worst-Case Early Lockdown | 1,701,684 | 250,507 | 6,642,557 | 6,893,064 | 8,594,748 | 1,923,385 | 18,534 |
| | Worst-Case Late Lockdown | 1,600,323 | 329,172 | 6,486,528 | 6,815,700 | 8,416,023 | 2,022,335 | 195,543 |
| Standard Policy 2 | Baseline Early Lockdown | 2,153,733 | 180,997 | 6,635,396 | 6,816,393 | 8,970,126 | 1,487,913 | 8,630 |
| | Baseline Late Lockdown | 1,891,369 | 201,292 | 6,642,348 | 6,843,640 | 8,735,009 | 1,742,580 | 9,526 |
| | Best-Case Early Lockdown | 3,849,717 | 182,036 | 4,317,975 | 4,500,011 | 8,349,728 | 0 | 0 |
| | Best-Case Late Lockdown | 3,841,505 | 202,363 | 4,325,468 | 4,527,831 | 8,369,336 | 673 | 0 |
| | Worst-Case Early Lockdown | 1,555,954 | 250,441 | 6,647,757 | 6,898,198 | 8,454,152 | 2,062,243 | 20,551 |
| | Worst-Case Late Lockdown | 1,471,078 | 328,614 | 6,488,018 | 6,816,632 | 8,287,710 | 2,145,029 | 201,798 |
| Standard Policy 3 | Baseline Early Lockdown | 2,159,482 | 180,831 | 6,630,895 | 6,811,726 | 8,971,208 | 1,484,984 | 10,428 |
| | Baseline Late Lockdown | 2,051,737 | 201,267 | 6,633,610 | 6,834,877 | 8,886,614 | 1,590,895 | 9,433 |
| | Best-Case Early Lockdown | 3,860,265 | 182,036 | 4,307,491 | 4,489,527 | 8,349,792 | 0 | 0 |
| | Best-Case Late Lockdown | 3,860,364 | 202,363 | 4,307,379 | 4,509,742 | 8,370,106 | 0 | 0 |
| | Worst-Case Early Lockdown | 1,755,576 | 250,134 | 6,632,584 | 6,882,718 | 8,638,294 | 1,875,503 | 22,830 |
| | Worst-Case Late Lockdown | 1,659,451 | 329,073 | 6,479,456 | 6,808,529 | 8,467,980 | 1,970,263 | 195,690 |
| Standard Policy 4 | Baseline Early Lockdown | 2,159,482 | 180,831 | 6,630,895 | 6,811,726 | 8,971,208 | 1,484,984 | 10,428 |
| | Baseline Late Lockdown | 1,977,949 | 200,981 | 6,632,999 | 6,833,980 | 8,811,929 | 1,663,329 | 11,854 |
| | Best-Case Early Lockdown | 3,860,265 | 182,036 | 4,307,491 | 4,489,527 | 8,349,792 | 0 | 0 |
| | Best-Case Late Lockdown | 3,856,966 | 202,363 | 4,310,748 | 4,513,111 | 8,370,077 | 0 | 0 |
| | Worst-Case Early Lockdown | 1,616,233 | 250,193 | 6,642,388 | 6,892,581 | 8,508,814 | 2,006,913 | 21,226 |
| | Worst- Case Late Lockdown | 1,559,665 | 328,743 | 6,480,892 | 6,809,635 | 8,369,300 | 2,064,784 | 200,491 |

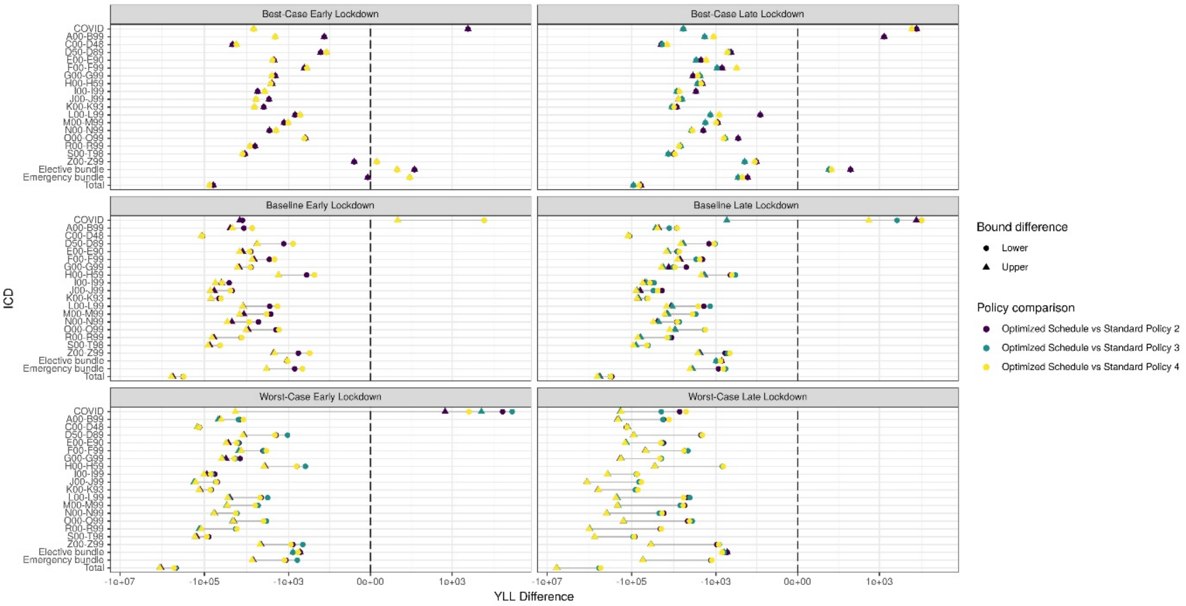

Supplementary Figure 6. Comparison of Standard Policies 2-4 with the Optimized Schedule for Years of Life Lost (YLL). The difference in YLL for all admissions under Standard Policies and Optimized Schedule ($YLL_{OS} - YLL_{SP}$) over the 52-week planning horizon. Log of difference presented.

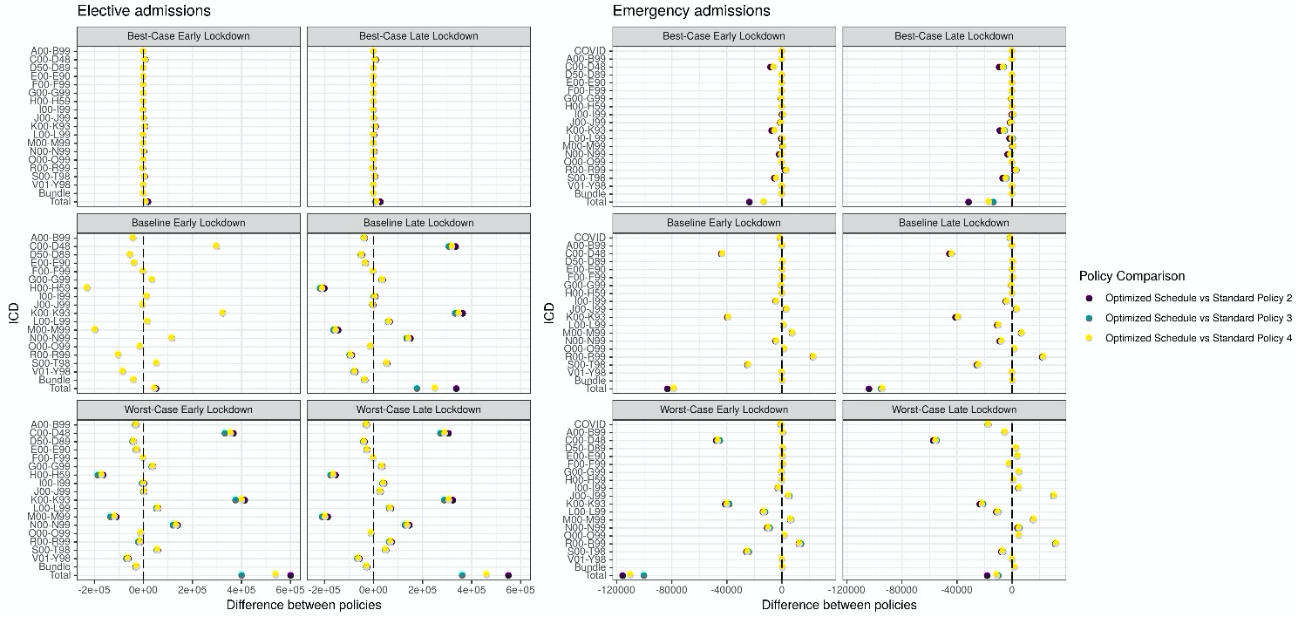


Supplementary Figure 7. Difference in Elective and Emergency Admissions between Optimized Schedule and Standard Policies 2-4, by ICD. Difference $= \text{Admissions}_{\text{OS}} - \text{Admissions}_{\text{SP}}$.

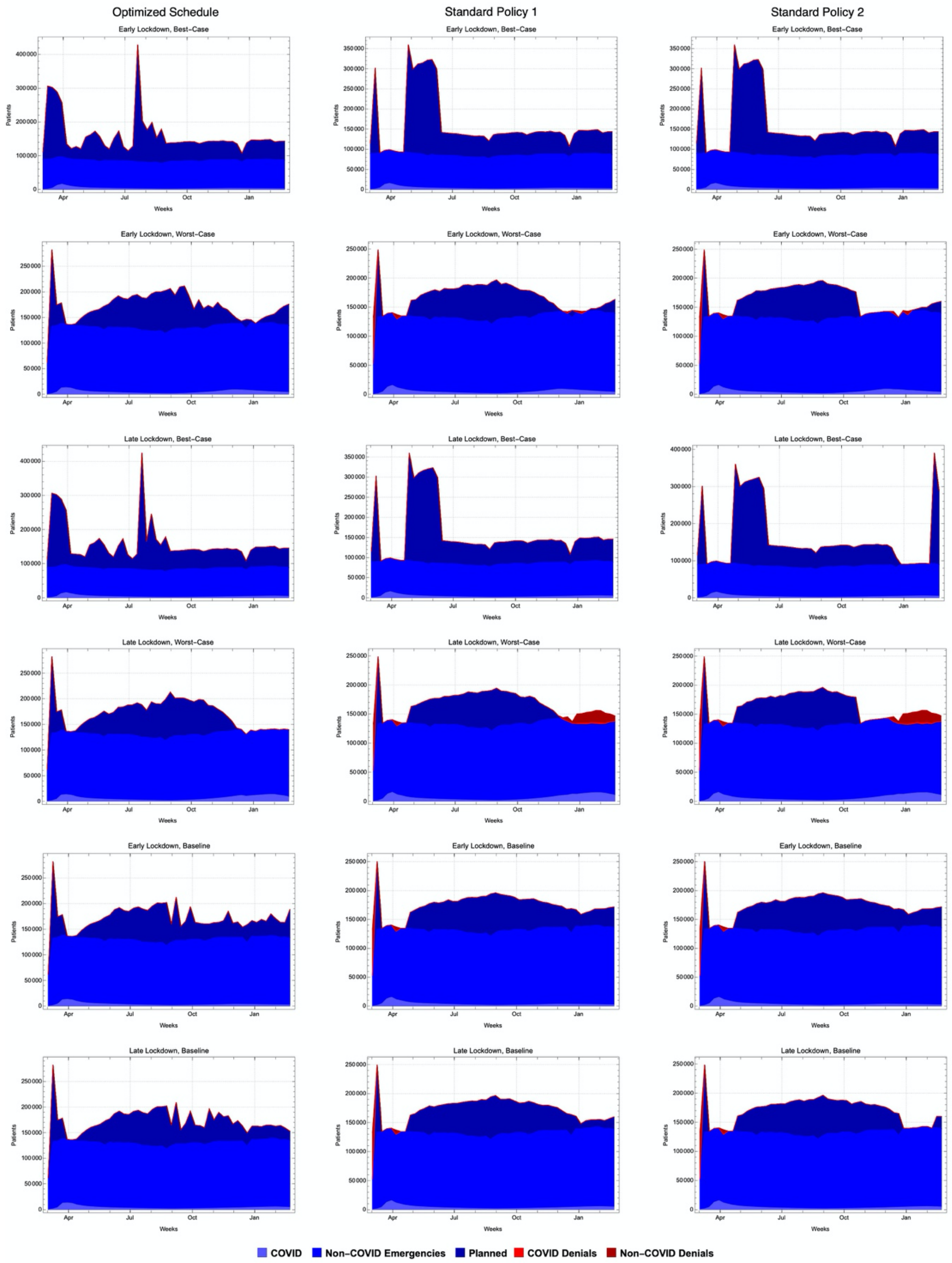


Supplementary Figure 8. Comparison of Standard Policies and Optimized Schedules for admissions and admission denials over the planning horizon

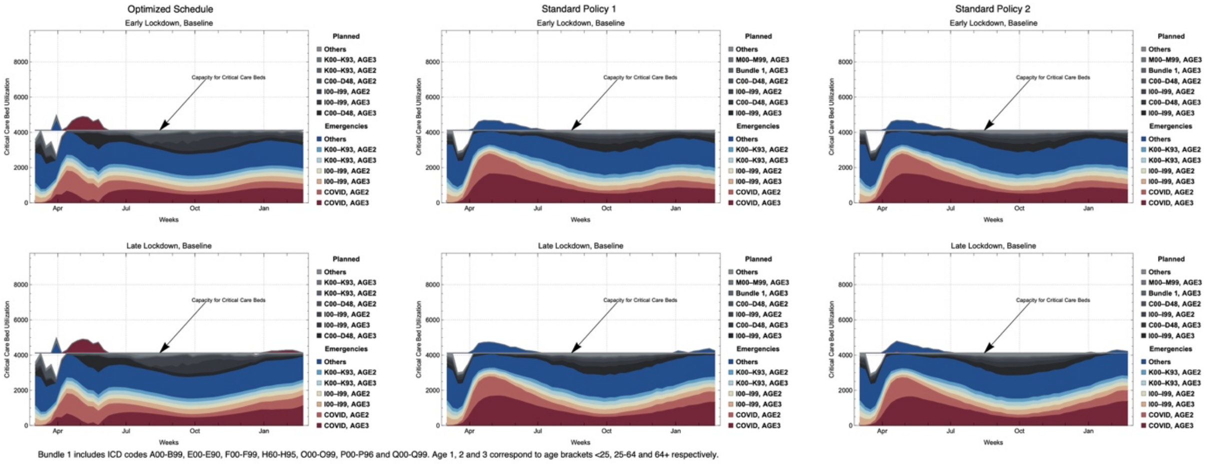

Supplementary Figure 9. Comparison of CC bed utilization between Optimized Schedules and Standard Policy scenarios for Baseline scenarios with Early and Late Lockdown

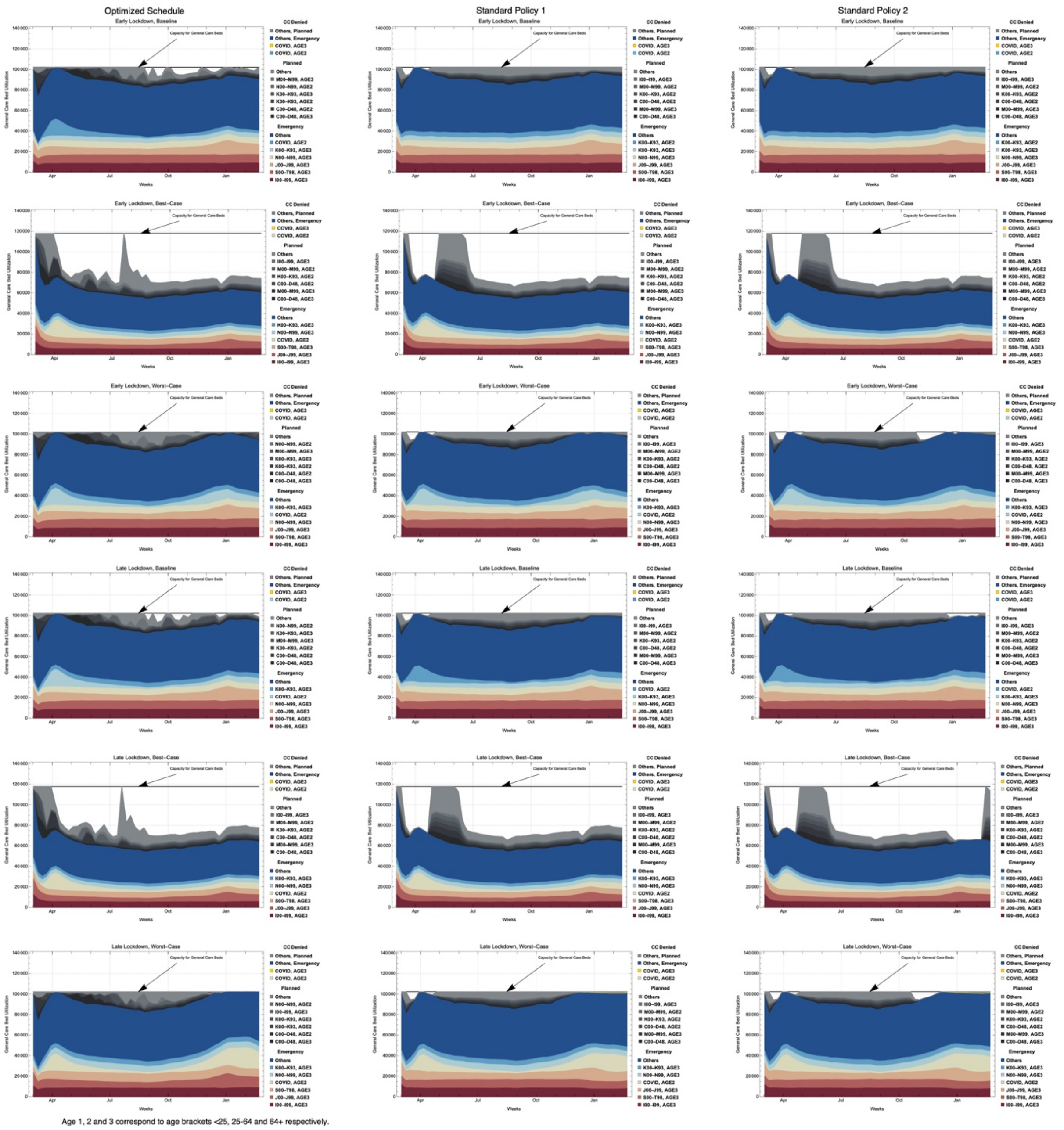


Supplementary Figure 10. Comparison of G&A bed utilization between Optimized Schedules and Standard Policy scenarios for Baseline, Best-Case and Worst-Case scenarios

**Supplementary Section 10 References**


1 D'Aeth J, Ghosal S, Grimm F, *et al.* Optimal Hospital Care Scheduling During the SARS-CoV-2 Pandemic. 2021.
2 Harvey AC. Forecasting, Structural Time Series Models and the Kalman Filter. Cambridge University Press, 1990 DOI:10.1017/CBO9781107049994.
3 Durbin J, Koopman SJ. Time Series Analysis by State Space Methods. Oxford University Press, 2012 DOI:10.1093/acprof:oso/9780199641178.001.0001.
4 Helske J. KFAS : Exponential Family State Space Models in R. *Journal of Statistical Software* 2017; **78**. DOI:10.18637/jss.v078.i10.
5 GitHub - khauck2606/DAEDALUS: An integrated economic-epidemiological model to project closure strategies differentiated by economic sector for maximizing economic production in the presence of SARS-CoV-2. .
6 GitHub - mrc-ide/sircovid. https://github.com/mrc-ide/sircovid (accessed Nov 15, 2020).
7 NHS Digital. Statistics » COVID-19 Hospital Activity. .
8 Office for National Statistics. Business Impact of COVID-19 Survey (BICS) results. .
9 Diekmann O, Heesterbeek JAP, Roberts MG. The construction of next-generation matrices for compartmental epidemic models. *Journal of The Royal Society Interface* 2010; **7**: 873–85.
10 Therneau TM. Survival Analysis [R package survival version 3.2-7]. 2020; published online Sept 28.
11 NHS Digital. HRG4+ 2020/21 Local Payment Grouper V2 (COVID-19). .
12 Office for National Statistics. Expectation of life, principal projection, UK. Office for National Statistics. .
13 National Institute for Excellence Health and Care Excellence (NICE). Assess frailty COVID-19 rapid guideline : critical care in adults. 2020.
14 Slides and datasets to accompany coronavirus press conference: 23 April 2020 - GOV.UK. https://www.gov.uk/government/publications/slides-and-datasets-to-accompany-coronavirus-press-conference-23-april-2020 (accessed Nov 15, 2020).
